# Combining Native Mass Spectrometry and Proteomics to Differentiate and Map the Metalloform Landscape in Metallothioneins

**DOI:** 10.1021/acs.jproteome.4c00271

**Published:** 2024-07-12

**Authors:** Manuel David Peris-Díaz, Alicja Orzeł, Sylwia Wu, Karolina Mosna, Perdita E. Barran, Artur Krężel

**Affiliations:** † Department of Chemical Biology, Faculty of Biotechnology, University of Wrocław, F. Joliot-Curie 14a, Wrocław 50-383, Poland; ‡ Michael Barber Centre for Collaborative Mass Spectrometry, Manchester Institute of Biotechnology, 131 Princess Street, Manchester M1 7DN, U.K.

**Keywords:** top-down mass spectrometry, native mass spectrometry, metallothionein, metal binding, metalloform, proteoform, collision induced unfolding

## Abstract

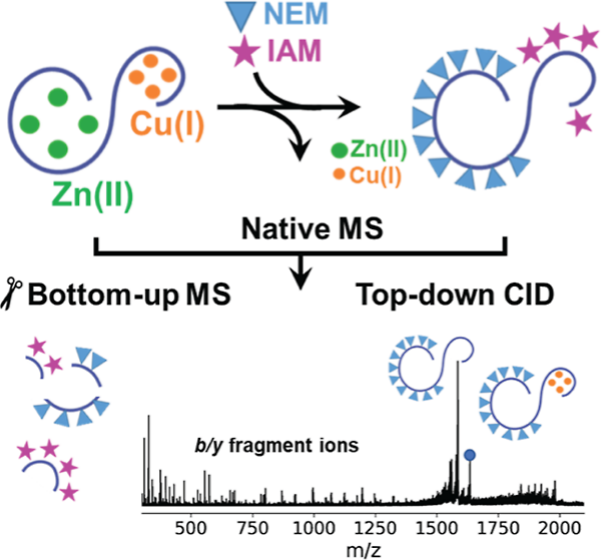

Within the intricate landscape of the proteome, approximately 30% of all proteins bind metal ions. This repertoire is even larger when considering all the different forms of a protein, known as proteoforms. Here, we propose the term “metalloforms” to refer to different structural or functional variations of a protein resulting from the binding of various hetero- or homogeneous metal ions. Using human Cu(I)/Zn(II)-metallothionein-3 as a representative model, we developed a chemical proteomics strategy to simultaneously differentiate and map Zn(II) and Cu(I) metal binding sites. In the first labeling step, *N*-ethylmaleimide reacts with Cysteine (Cys), resulting in the dissociation of all Zn(II) ions while Cu(I) remains bound to the protein. In the second labeling step, iodoacetamide is utilized to label Cu(I)-bound Cys residues. Native mass spectrometry (MS) was used to determine the metal/labeling protein stoichiometries, while bottom-up/top-down MS was used to map the Cys-labeled residues. Next, we used a developed methodology to interrogate an isolated rabbit liver metallothionein fraction containing three metallothionein-2 isoforms and multiple Cd(II)/Zn(II) metalloforms. The approach detailed in this study thus holds the potential to decode the metalloproteoform diversity within other proteins.

## Introduction

Approximately 30% of human genes encode proteins that bind metal ions as cofactors to catalyze reactions, stabilize protein structures, or regulate total or mobile metal concentrations.^1^ Those estimations consider only a single existing state of a protein, whereas in reality, a protein can exist in multiple states, collectively known as proteoforms.^2^ Among all essential metals in mammals, Zn(II), Fe(II)/(III), and Cu(I)/(II) are critical for enormous physiological functions.^3^ The proper regulation of these metal ions is crucial to the overall homeostatic stability of an organism. Mammalian metallothioneins (MTs) play a crucial role in maintaining the homeostatic control of Zn(II) and Cu(I) ions in cells, ensuring the proper balance and regulation of these metal ions within cellular environments.^4,5^ They constitute a family of low-molecular-weight, cysteine-rich proteins comprising at least a dozen MT isoforms (MT1–4 isoforms and multiple subisoforms), which vary in sequence, tissue localization, function specificity, and metal binding properties.^4,6−8^ Among them, metallothionein-3 (MT3), primarily expressed in the central nervous system (CNS), plays a crucial role in the regulation of Zn(II) trafficking and acts as a protective mechanism against neurodegenerative disorders by replacing Zn(II) with excessive copper [Cu(II)/Cu(I)] in the brain.^9−12^ As a result of this biological process, MT3 exists in multiple heterogeneous and homogeneous Cu(I)/Zn(II) states, referred to as metalloforms.

Cysteine, the most nucleophilic amino acid residue in proteins, is a target for reactive oxygen, nitrogen, and sulfur species and numerous chemical reactions, including post-translational, and critical for binding essential and toxic metal ions.^13−15^ Recognizing the importance of Cys residues, a variety of experimental and theoretical tools have been developed to investigate the different redox states of Cys residues within proteomes, enabling a deeper understanding of their functional implications.^16−19^ The detection and analysis of cysteine residues often rely on using thiol-specific probes that react with the nucleophilic sulfur of cysteine.^20,21^ These probes exhibit different reactivity profiles, allowing for the differentiation of various forms and states of cysteine. Commonly employed protein thiol probes include iodoacetamide (IAM), iodoacetic acid (IAA), *N*-ethylmaleimide (NEM), methylmethanethiosulfonate (MMTS), Cys-reactive mass tag (cys-TMT), and *p*-benzoquinone (Bq).^22,23^ IAM and NEM are widely used probes that covalently react with sulfhydryl groups using different mechanisms.^13,24^ IAM undergoes S_N_2 nucleophilic substitution, while NEM proceeds via nucleophilic Michael addition. NEM exhibits a faster reaction rate with thiols than IAM and is effective over a wider pH range, including acidic conditions.^24^ In contrast, IAM requires neutral or basic pH.^20^ However, NEM shows lower specificity, potentially leading to side reactions with histidine and lysine residues at high excess or basic pH. IAM is preferred due to its formation of stable thioether bonds, while NEM may undergo partial ring hydrolysis. These alkylation reagents have been independently used in the past to study homogeneous metal complexes in metallothioneins.^25−32^ Another experimental approach involves using differential alkylation techniques to map cysteine redox states in purified proteins and cellular proteomes.^19,33−35^ This method involves the initial blocking of a reduced free thiol using one alkylator, followed by a reduction step and subsequent labeling using a different alkylator. By employing this approach, cysteine redox modifications can be identified and characterized, providing insights into the redox status of cysteine residues. These chemical methodologies are usually coupled with high-resolution mass spectrometry (MS). Our previous research presented a differential labeling approach capable of distinguishing between free Cys and Zn(II)-protected Cys residues within zinc sites, exhibiting nanomolar and picomolar metal affinities.^36^ This methodology has been used to demonstrate the absence of the Zn(II) ion in alcohol dehydrogenase 1 in a murine model of alcohol-associated liver disease.^19^

Here, we developed an MS-based differential alkylation strategy to distinguish and map metalloform landscapes. As a representative model protein, we selected heterogeneous Cu(I)/Zn(II)-MT3, which has been previously demonstrated to exist as an air-stable Cu(I)_4_Zn(II)_3–4_MT3 complex when isolated from mammalian brains.^37,38^ MT3 folds in a dumbbell-shaped structure comprising 68 amino acid residues and encompassing two domains.^39,40^ Spectroscopic studies have unveiled the interaction of MT3 with divalent metal ions (M), resulting in the formation of an M_3_Cys_9_ metal cluster in the N-terminal β-domain and an M_4_Cys_11_ metal cluster in the C-terminal α-domain.^8,41,42^ In vitro investigations have provided evidence that Zn_7_MT3 reacts with Cu(I)/Cu(II) ions, involving Zn(II)-to-Cu(I) exchange, Cu(II) reduction to Cu(I), and the formation of intramolecular disulfide bonds, resulting in the formation of Cu(I)_4_Zn(II)_4_MT3_ox_.^43−46^ Interestingly, no preference for a specific domain was found when using Cu(I) titrations on Zn_7_MT3.^47,48^ Using this protein, we demonstrated that NEM could be employed in the initial labeling step to dissociate all Zn(II) ions while Cu(I) remains bound due to its higher affinity for Cys. In the second labeling step, an excess of IAM is utilized to label Cu(I)-bound Cys residues, leading to Cu(I) dissociation. Native MS is used to monitor the reaction at each step, and bottom-up/top-down MS techniques are utilized to map the Cys-IAM and Cys-NEM labeled residues, allowing for the determination of the locations of Cu(I) and Zn(II) within the protein, respectively. The developed methodology was then tested on an isolated rabbit liver fraction containing N-acetylated metallothionein-2a/b/c isoforms. NEM was used to dissociate Zn(II) ions, while Cd(II) remained bound to the protein. Next, IAM was employed to dissociate tightly bound Cd(II) ions and label the Cys residues. Combining native MS with proteomics approaches unveiled the location of Zn(II) and Cd(II) ions in all metallothionein-2 isoforms. The described methodology has the potential to be expanded for the investigation of metalloforms.

## Experimental Section

Metallothionein-3 (MT3) (Addgene plasmid ID 105710) was overexpressed and purified in a bacterial system as described in the Supporting Information. Rabbit liver metallothionein-2 (MT2) was purchased from Santa Cruz Biotechnology, Inc., USA. The preparation of the samples for the experiments is described in the Supporting Information. Electronic spectroscopy experiments were performed on a JASCO V-750 spectrophotometer, at 25 °C, in a 1 cm quartz cuvette, as described in the Supporting Information.

MS experiments were carried out on a Synapt XS HDMS equipped with nanoelectrospray ionization (Waters Corp., UK) using a 500–5000 *m*/*z* range. 5–10 μL of the sample (10–20 μM in 200 mM ammonium acetate) was loaded into borosilicate glass capillaries (O.D. 1.2 mm, I.D. 0.9 mm, World Precision Instruments, Stevenage, UK) produced in-house using a Flaming/Brown P-1000 micropipette puller (Sutter Instrument Co., Novato, CA, USA) and ions were produced by applying a positive potential of 0.9–1.4 kV via a platinum wire (Goodfellow).

Native top-down collision-induced dissociation (CID) MS experiments were performed by applying 20–60 V of trap collision energies of quadrupole-selected ions with argon as the collision gas. Detailed calculation description of the survival yield (SY) curves and data analysis can be found in the Supporting Information. Top-down electron transfer dissociation (ETD) MS experiments were performed by introducing the sample via syringe with a 2–3 μL·min^–1^ flow rate and a spray voltage of 2–2.5 kV. The glow discharge was tuned to obtain an ETD reagent (1,4-dicyanobenzene) current of ∼1 × 10^6^ counts/s for charge reduction. The anions were accumulated in the trap collision cell for 100 ms using a refill interval of 1 s. The reaction was started by lowering the wave height from 1.5 to 0.2–0.3 V, using a wave velocity of 300 ms^–1^.

To perform bottom-up MS experiments, single or dual labeled MT3 proteins were buffer exchanged to 100 mM ammonium bicarbonate. Trypsin (Sigma-Aldrich) was added at 1:1 (w/w), followed by the addition of 0.1% RapiGest SF (Waters Corp., UK) and overnight digestion at 37 °C. Samples were acidified to 0.5% formic acid (FA) to quench digestion, centrifuged, and desalted on Stage Tips tips eluting the peptides with 80:20 ACN/H_2_O 0.1% FA. After removing ACN (acetonitrile) by speed-vac, the peptides were resuspended in 0.1% FA. Samples were analyzed via LC–MS using a Waters Acquity UPLC m-class system coupled to a Synapt XS HDMS operated in a positive ion/resolution mode. Peptides were first trapped on a Waters Acquity BEH C18 1.7 μm VANGUARD column and then separated on a Waters Acquity UPLC BEH C18 1.7 μm, 1.0 × 100 mm. Mobile phase A consisted of 0.1% FA (v/v) in Milli-Q water, while mobile phase B consisted of 0.1% FA (v/v) in ACN. The LC gradient was supplied at 40 μL·min^–1^ over a 22 min gradient (5–35% B). MS analysis was performed using MS^e^ data independent acquisition, which used a collision energy ramp from 20 to 45 V in a 50–2000 *m*/*z* range for the high energy scans. Raw LC-MS^e^ data files were processed using a ProteinLynx Global Server 3.0.3 (Waters Corp., UK) and searched against the MT3 fasta protein sequence (UniProt P25713). The search was performed using 15 ppm as a precursor mass tolerance and a fragment mass tolerance of 20 ppm with three minimum fragment ion matches per peptide. Trypsin was set as the protease with a maximum of three missed cleavage allowed. Cysteine carbamidomethyl, cystine, and Cysteine *N*-ethylmaleimide were set as variable modifications. Leu-Enkephalin (556.27 *m*/*z*) was used as a lock-mass. Peak lists were exported as text files and analyzed using custom scripts in Python 3.5. Native MS and top-down MS experiments were analyzed using MassLynx v4.2 (Waters Corp., UK) and custom scripts in Python 3.5 (available at https://github.com/ManuelPerisDiaz/Cu-Zn-MT3) as described in the Supporting Information.

## Results and Discussion

In order to develop a differential labeling strategy that may selectively target Cys residues in proteins that bind Zn(II) and Cu(I) metal ions, we first prepare the heterogeneous Cu(I)_4_Zn(II)_4_MT3_ox_ complex. Addition of four CuCl_2_ molar equivalent (mol Eq) to Zn_7_MT3_red_ (reduced and fully Zn(II)-loaded MT3) results in the formation of the Cu(I)_4_Zn(II)_4_MT3_ox_ along with other metal complexes.

**Figure 1:**
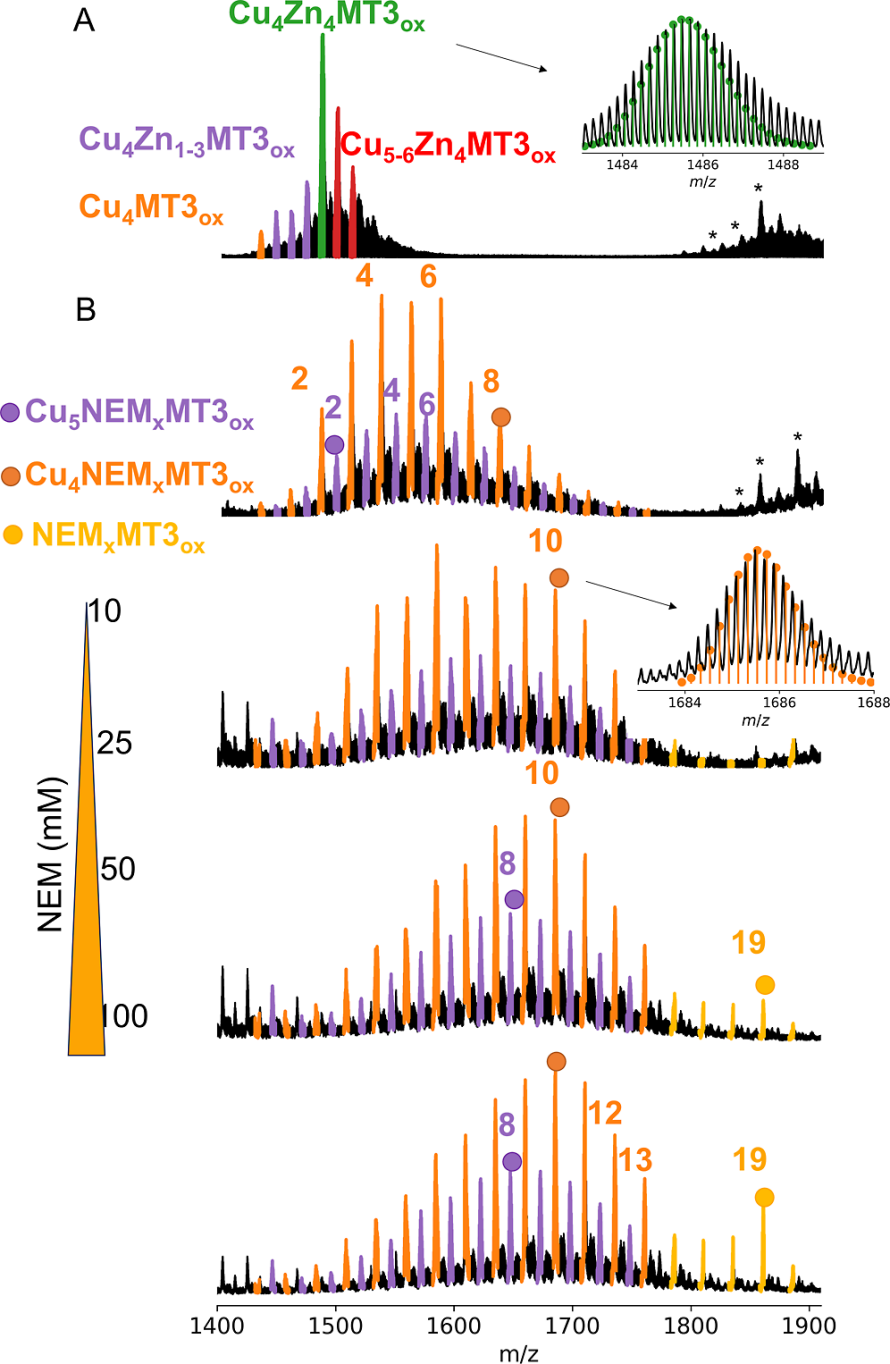
Native mass spectra of the Cu(I)/Zn(II)-MT3_ox_ complexes obtained after incubation of Zn_7_MT3_red_ with 4 CuCl_2_ mol eq at increasing concentration of NEM. The *m*/*z* region corresponds to 5+ ions (full spectra in Figure S5). The proteins (10 μM) were sprayed in 200 mM ammonium acetate (pH 6.8). “ox” subscript refers to oxidized (two intramolecular disulfides) MT3. Peak series corresponding to Cu_4_NEM_0–13_MT3_ox_ are colored in orange, while peak series corresponding to Cu_5_NEM_0–12_MT3_ox_ are colored in violet. The asterisks indicate *m*/*z* signals that correspond to 4+ ions.

We observed at least seven metalloforms (Cu_4_MT3_ox_, Cu_4_Zn_1_MT3_ox_, Cu_4_Zn_2_MT3_ox_, Cu_4_Zn_3_MT3_ox_, Cu_4_Zn_4_MT3_ox_, Cu_5_Zn_4_MT3_ox_, and Cu_6_Zn_4_MT3_ox_). The mechanism of the reaction involves a Zn(II)-to-Cu(I) exchange by reducing Cu(II) to Cu(I) through the formation of two intramolecular disulfide bonds (indicated here as “ox”) (Figures 1 and S5). To precisely annotate the ions to particular complexes, multiple theoretical isotopic distributions for the protein were generated, and the one demonstrating the optimal fit was selected as outlined in Figure S1. We observed all series from tetra-to heptametallic complexes, which suggests that the reaction proceeds by first Zn(II) clusters disassembly (Zn_3_S_9_ and Zn_4_S_11_) and then reassembly following a probability distribution. Monitoring intensities of the ligand-to-metal charge transfer (LMCT) transition in the middle-to-far UV range also showed that Zn_7_MT3_red_ is able to bind and reduce Cu(II) to Cu(I) with concomitant Zn(II) displacement and disulfides’ formation (Figure S2).

### Profiling Reactive Cysteine Residues in Cu(I)/Zn(II)-MT3_ox_ Species by NEM and IAM

**Scheme 1:**
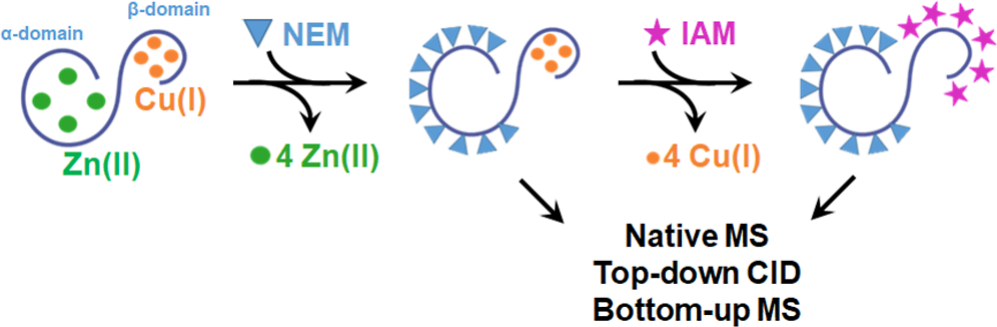
Overview of the Presented Differential Labeling Strategy for Labeling Zn(II)- and Cu(I)-Cys Metal Binding Sites

Once we had prepared the heterogeneous Cu(I)_4_Zn(II)_4_MT3_ox_ complex, we studied the kinetic and thermodynamic lability of the metal–thiolate bonds by using the cysteine alkylators IAM and NEM, which follow a different reaction chemistry. While IAM follows a nucleophilic substitution S_N_2, NEM reacts following a nucleophilic addition. To do so, the metal-protein complex was incubated with increasing concentrations of both alkylation reagents for different periods of time, and the reaction was monitored by native MS. The addition of IAM to the cocktail of Cu(I)/Zn(II)-MT3 complexes formed upon Cu(II) reaction with Zn_7_MT3_red_ results in a mixture of Cu(I)_*x*_Zn(II)_*y*_IAM_*z*_MT3_ox_ species, coexisting simultaneously (Figure S3A). In our previous paper, we reported that IAM is a very good choice not only for labeling free Cys residues but also for mapping Zn(II) binding sites. Its low reactivity prevents it from competing with Zn(II) ions for Cys residues.^36^ As we have partially metal-loaded species upon Cu(II) reaction with Zn_7_MT3_red,_ IAM reacts with free Cys residues in the Cu_4_Zn_1–3_MT3_ox_ complex as well as Cu_4_MT3_ox_. Then, the use of IAM leads to the stepwise dissociation of four Zn(II) ions in Cu_4_Zn_4_MT3_ox_ and Cu_5–6_Zn_4_MT3_ox_, and finally, at high concentrations (100 mM), IAM can react with Cu(I)-Cys bound residues, dissociating the Cu(I) ions (Figure S3B). However, at different combinations of IAM concentration and reaction time, we have multiple and heterogeneous Cu(I)/Zn(II)-MT3 complexes, which can be very difficult to interrogate by using a well-established bottom-up proteomics approach. As the main aim is to develop a differential labeling strategy to map Cu(I)/Zn(II) binding sites in proteins, we then decided to use NEM. We have shown that NEM reacts cooperatively and fast with Cys residues that had bound Zn(II) with weak and moderate binding affinities. Even at low NEM concentrations, we clearly observe selective dissociation of most of the Zn(II) ions while keeping Cu(I) bound to the protein (Figures 1 and S4 and S5). Increasing NEM concentration results in complete dissociation of Zn(II), obtaining products that only contain four and five Cu(I) ions, and a different extent of NEM modification. Such results highlight that NEM produces homogeneous Cu(I)-MT3_ox_ complexes and could ease the identification and conclusions about NEM moieties using a bottom-up MS approach (Scheme 1). We could observe that the modification profile was centered at Cu(I)_5_NEM_7–9_MT3_ox_ and Cu(I)_4_NEM_8–12_MT3_ox_ species, and these remain the most intense ions upon doubling the NEM concentration. The difference in two NEM moieties between both proteoform families arises from the coordination chemistry, as Cu(I) tends to bind to Cys residues forming a linear geometry. The NEM experiments revealed that out of 20 Cys residues in the MT3 protein, 13 Cys residues could be modified by NEM, while the four Cu(I) ions were bound to MT3 (Figures 1 and S4).

### CID Experiments on Cu(I)/Zn(II)-NEM_*x*_MT3_ox_ Species

**Figure 2:**
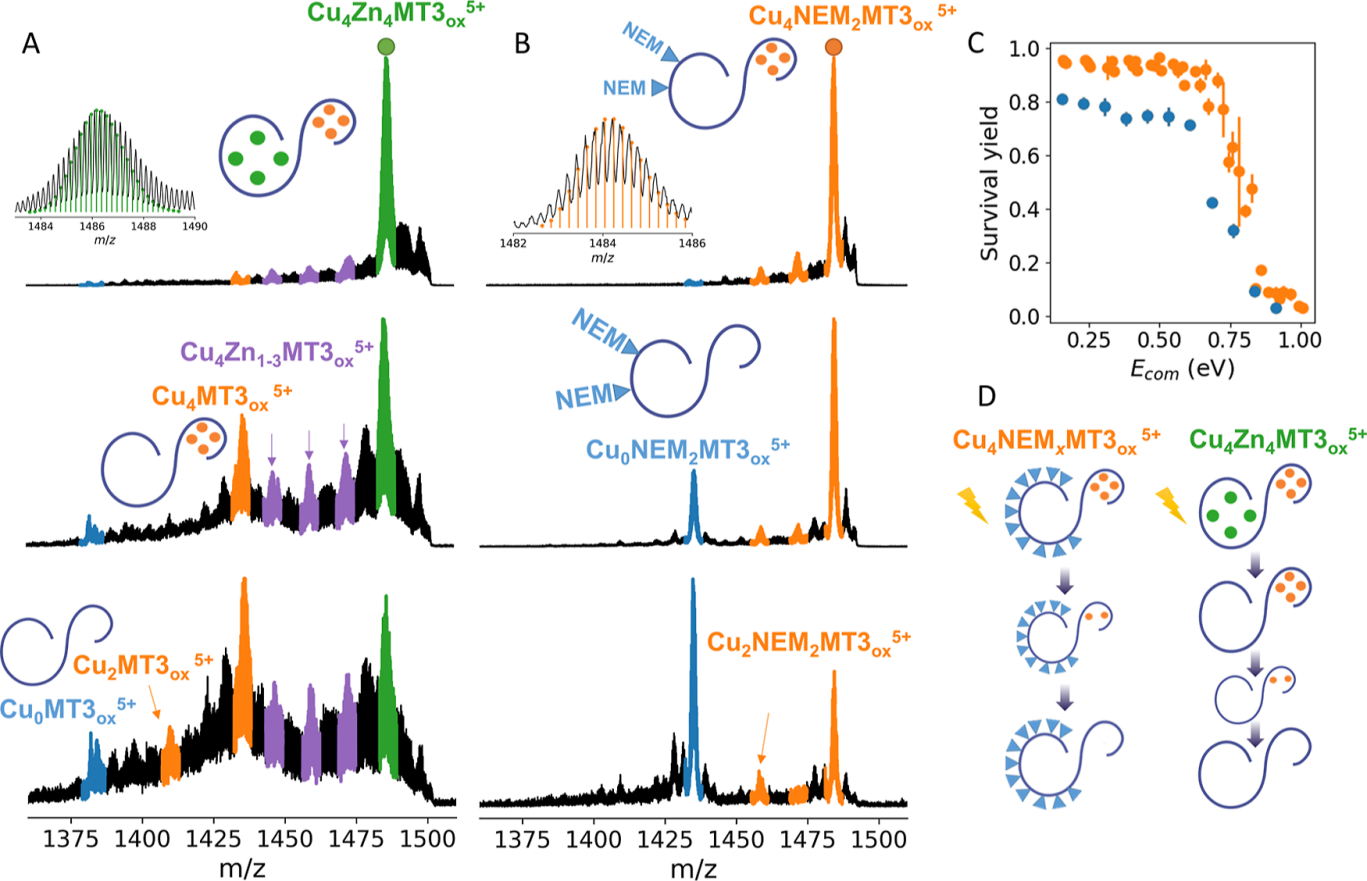
CID experiments. Mass spectra acquired under different collision energies (10, 50, and 60 eV) for quadrupole-selected Cu_4_Zn_4_MT3_ox_^5+^ (A) and Cu_4_NEM_2_MT3_ox_^5+^ (B). SY plots for quadrupole-selected Cu_4_NEM_2–13_MT3_ox_^5+^ (orange) and for NEM_18_MT3_ox_^5+^ (blue) (C). Schematic representation of the metal ion dissociation mechanism inferred from CID experiments (D). “*E*_com_” denotes the center-of-mass energies as defined in the Supporting Information. Figure S7 shows the isotopic distributions for each ion and its assignment based on theoretical distributions.

To identify the ligands bound to MT3 and confirm the stoichiometries inferred from the native MS experiments, we employed a native top-down MS approach. First, we mass-selected the Cu(I)_4_Zn(II)_4_MT3_ox_^5+^ ions and subjected them to CID. The CID spectra contain an *m*/*z* region, with ions corresponding to the precursor and the precursor with the loss of some metal ions, thus providing direct information about the number and nature of metal ions bound to the protein (Figure S6). Under collisional activation (CA) conditions, we observed a stepwise dissociation of the Zn(II) ions from Cu(I)_4_Zn(II)_4_MT3_ox_^5+^ to form an intermediate of Cu(I)_4_MT3_ox_^5+^ ions (Figure 2A). The signals were isotopically resolved and therefore permitted us to observe that increasing CA leads to a cooperative dissociation of the four Cu(I) ions from Cu(I)_4_MT3_ox_^5+^ to form Cu(I)_0_MT3_ox_^5+^ (Figure S7). This experiment demonstrates that CA can be used to induce metal ion dissociation and, therefore, identify the ligands that bind to proteins. Moreover, it is evident here that applying CA to intact metal-protein complexes is not the best choice for mapping metal binding sites, as we obtain partial metal ion dissociation. One strategy to avoid this issue is the use of Cys alkylators. However, the unambiguous determination of alkylator and metal protein stoichiometries is not always trivial due to the close masses. By applying the CID approach, we can further identify the precise Cu(I):NEM:MT3 stoichiometries (Figure 2B). For example, after adding 10 mM NEM to the mixture of Cu(I)/Zn(II)-MT3 complexes, namely, Cu_4_MT3_ox_, Cu_4_Zn_1–3_MT3_ox_, Cu_4_Zn_4_MT3_ox_, and Cu_5–6_Zn_4_MT3_ox_, some signals could be annotated based on accurate mass. However, for others, multiple stoichiometries could be annotated, as illustrated by the 1486 *m*/*z* signal (Figures 1 and 2A). We observed a signal that overlaps in the same *m*/*z* range (∼1486 *m*/*z*) as the Cu(I)_4_Zn(II)_4_MT3_ox_^5+^ ions after NEM addition. Subjecting these ions to CID activation revealed the precise stoichiometry of Cu(I) ions and NEM moieties, namely, Cu(I)_4_NEM_2_MT3_ox_^5+^ (Figure 2B). This strategy was also applied to the signals annotated with five Cu(I) ions, and we observed the dissociation of the five Cu(I) ions upon CID activation (Figure S8).

The gradual increase in kinetic energy also allowed us to examine the stability of the Cu(I)-thiolate bonds. Isolation and soft activation of the Cu(I)_4_NEM_2_MT3_ox_^5+^ ions led to the appearance of signals corresponding to Cu(I)_3_NEM_2_MT3_ox_^5+^, which, upon harder ion activation, lost one Cu(I) ion, yielding Cu(I)_2_NEM_2_MT3_ox_^5+^ ions (Figure 2B). Such a binuclear Cu(I) intermediate was found in all of the Cu(I)_4_NEM_2–14_MT3 complexes after CID activation (Figures S9 and 2). These results indicate a change in the metal cluster structure from a well-defined Cu(I)_4_-thiolate to a favored binuclear Cu(I)-thiolate. We then estimated the different relative ion stabilities of the Cu(I)_4_NEM_*x*_MT3_ox_^5+^ protein ion complexes by calculating SY plots (Figure S10).^49^ They indicate that all of the Cu(I)_4_NEM_*x*_MT3_ox_^5+^ ions show a similar stability (0.79–0.81 eV) and are more stable than NEM_18_MT3_ox_^5+^ ions (0.72 eV), revealing the stabilizing role of the Cu(I)_4_-thiolate cluster in the protein structure (Figure 2C,D).

### Native Top-Down and Bottom-Up MS of Cu(I)_4_NEM_*x*_MT3_ox_ Species

**Figure 3:**
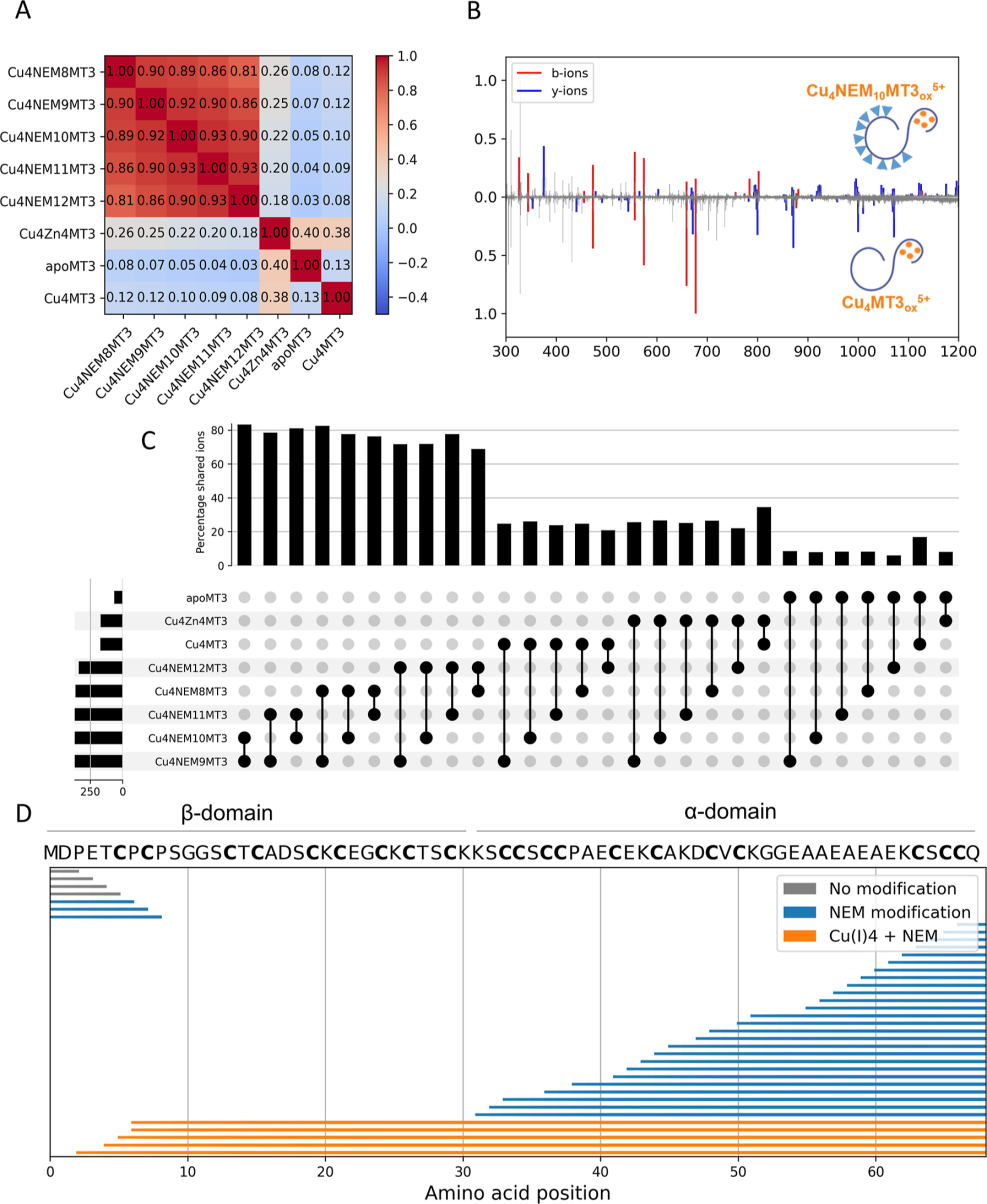
Native top-down CID MS. Correlation matrix based on peak intensities of the fragmentation spectrum (A). Mirror fragmentation plot acquired for quadrupole-selected Cu_4_NEM_10_MT3_ox_^5+^ and Cu_4_MT3_ox_^5+^ (B). Upset plot analysis of all Cu(I)_4_NEM_8–12_MT3_ox_^5+^, Cu_4_MT3_ox_^5+^, Zn_4_Cu_4_MT3_ox_^5+^, and apoMT3_ox_^5+^. The horizontal bar plots show the number of detected peaks for each fragmentation spectra, and each vertical bar plots the percentage of shared detected ions among two fragmentation spectra (C). CID fragmentation map of Cu(I)_4_NEM_10_MT3_ox_^5+^ (D).

As NEM remains bound to the protein upon CID activation, we could use a native top-down MS approach to localize which Cys residues are modified and, therefore, infer where Zn(II) ions were previously bound in the Cu(I)_4_Zn(II)_4_MT3_ox_ complex (Scheme 1). To this end, we mass-selected and fragmented each of the Cu(I)_4_NEM_*x*_MT3_ox_^5+^ (*x* = 8–12) ions as the NEM modification profile was centered around these species (Figures 1 and S11 and S12). The CID spectrum revealed three regions: a metal-free *b/y* fragment ion in the 300–800 *m*/*z* region, metal-free *y*-fragment ions in the 800–1200 *m*/*z* region, and *y*-fragment ions with four Cu(I) ions bound, observed at 1700–2000 *m*/*z* (Figure S12). To compare all fragmentation spectra, we calculated a correlation matrix based on ion intensities (Figure 3A). We observed a high correlation between the fragmentation patterns of all Cu(I)_4_NEM_*x*_MT3_ox_^5+^ (*x* = 8–12) complexes, with the similarity decreasing as the NEM stoichiometry increased. As a control to ensure that NEM alkylation did not induce Cu(I) migration to non-native metal binding sites, we obtained a CID spectrum of Cu(I)_4_MT3_ox_^5+^ (Figure 3B). A low correlation coefficient (*R* = 0.08–0.12) between the spectra of Cu(I)_4_MT3_ox_^5+^ and all Cu(I)_4_NEM_*x*_MT3_ox_^5+^ (*x* = 8–12) complexes was obtained (Figure 3A).

A more comprehensive analysis of the fragmentation spectrum that avoids using peak intensities was conducted using an UpSet plot analysis (Figure 3C). This analysis allowed us to calculate the percentage of detected fragment ions common among all fragmentation spectra. In agreement with the correlation analysis, the Cu(I)_4_NEM_*x*_MT3_ox_^5+^ (*x* = 8–12) complexes shared ca. 70–80% of the detected peaks and displayed high similarity. By employing this analysis, we demonstrated that ca. 30% of the ions were shared between Cu(I)_4_MT3_ox_^5+^ and/or Cu(I)_4_Zn(II)_4_MT3_ox_^5+^ complexes with Cu(I)_4_NEM_*x*_MT3_ox_^5+^ (*x* = 8–12) complexes.

In the next step, we annotated the previously detected peaks and observed that 75% of *b*-fragment ions were shared between both protein species. For Cu(I)_4_MT3_ox_^5+^, the Cu(I)-thiolate protects against generation of *b*-fragment ions, resulting in the annotation of only b2 to b8 fragments. The metal-free α-domain is easily fragmented upon CA, generating a series of *y*-fragment ions. For Cu(I)_4_NEM_10_MT3_ox_^5+^, we also annotated unmodified b2 to b5 fragment ions as well as specific b6 + 1NEM, b7 + 1NEM, and b8 + 2NEM fragment ions.

Similarly to Cu(I)_4_MT3_ox_^5+^, the metal-free α-domain was completely fragmented but generated NEM-labeled *y*-fragment ions (Figure 3B). This fragmentation pattern indicates that Cu(I) binding remains at the native sites after the NEM reaction. A close examination of the annotated fragments allowed us to conclude that the α-domain is metal-free, and the four Cu(I) ions are bound in the β–domain (Figure 3D). When using Cu(I) as a metal source rather than Cu(II), no domain preference was observed.^47,48^ Therefore, these results indicate that the intramolecular disulfide formation formed upon Cu(II) reduction to Cu(I) dictates the domain preference and not the nature of the metal ion.

Due to the low sequence coverage in the N-terminus β–domain, covering until the b8 fragment ion, one might suspect that this is due to metal protection by the Cu(I) ions. However, we have previously shown that even in apoMT3, complete fragmentation of the β–domain is not possible using CA.^50^ Our conclusions are based on obtaining almost a complete sequence coverage of *y*-ions spanning the α-domain, which exhibited varying degrees of NEM modification, up to 7 NEM moieties (y35 + 7NEM). Additionally, when considering the b8 + 2NEM fragment ion, we accounted for 9 out of the 10 NEM modifications observed in the Cu(I)_4_NEM_10_MT3_ox_ parent ion. These results indicate that Cys6 and Cys8 in the β–domain weakly interacts with Cu(I) ions, as they could be labeled by NEM. Two *b*-type fragment ions were found, matching with two proteoforms. A proteoform with Cys5 unmodified and a second proteoform with Cys5 and Cys7-NEM modified. Similarly, the α-domain does not bind Cu(I) ions either. By using this native top-down MS approach, we were able to determine the locations of several Cys-NEM labeled residues, such as Cys66, Cys67, and Cys64. In addition, we reconstructed the metal and NEM stoichiometries of the parent ion Cu(I)_4_NEM_10_MT3_ox_ by combining two fragment ions: y62 + 9NEM and b6 + 1NEM. In another attempt to localize the remaining Cys-NEM labeled residues, we performed top-down ETD (Figure S13). However, instead of obtaining ETD fragmentation, we observed a nondissociative electron transfer dissociation (ETnoD).^51^

To determine the locations of all NEM moieties in the Cu(I)_4_NEM_*x*_MT3_ox_^5+^ (*x* = 8–12) complexes, we initially prepared the Cu(I)/Zn(II)-MT3 as described above. Afterward, we added 25 mM NEM to the resulting mixture, and the sample was digested into peptides. These resulting peptides were then analyzed by bottom-up liquid chromatography–MS (nanoLC-MS/MS). The data revealed complete sequence coverage of Cys-NEM labeled residues from peptides covering the β–domain (residues 1–31) and α-domain (residues 32–68) (Figure S14A). This can be explained by the observation that, even though NEM produces metal homogeneous Cu(I)_4_NEM_*x*_MT3_ox_^5+^ (*x* = 8–12) complexes, portions of NEM_16–20_MT3_ox_^5+^ ions remain present upon reaction (Figure 1). Additionally, in a bottom-up MS, the peptide-to-proteoform connectivity is lost, and we cannot infer the origin of the peptide.^52^ Based on our recent findings that the Cu(I)_4_-thiolate cluster remains stable even under denaturing conditions, we decided to skip any desalting/purification step after enzymatic digestion, and the resulting peptide mixture was directly infused into the mass spectrometer.^53^ The recorded peptide mass fingerprint similarly displayed all Cys-NEM labeled residues, but we were also able to observe three peptides that covered the full β-domain (residues 1–31) and were bound to four Cu(I) ions (Figure S14B). Remarkably, these peptides also had two NEM moieties bound, consistent with our top-down MS analysis, which revealed that the β-domain binds four Cu(I) ions and that the Cys6 and Cys8 residues are labeled by NEM. Previous low-temperature (77 K) luminescence spectroscopy data identified 5–6 thiolate-Cu(I) bonds in the Cu(I)_4_Zn(II)_4_MT3 complex, suggesting the formation of a Cu(I)_4_Cys_5_ cluster and two intramolecular disulfides.^44^ Our high-resolution native MS data support the presence of two intramolecular disulfides in the Cu(I)_4_Zn(II)_4_MT3 complex (inset of Figure 1A), but they also reveal a population with only one disulfide. This matches our observations of two *b*-type proteoforms. Considering that four Cu(I) ions are bound in the β-domain, which contains 9 Cys residues, and that Cys6 and Cys8 are found both modified and unmodified, we conclude that Cu(I)_4_Cys_5_ cluster and two disulfides, involving Cys6 and Cys8, are formed. The location of second disulfide remains to be elucidated.

### Native Top-Down and Bottom-Up MS of NEM_*x*_IAM_*y*_MT3_ox_ Species

**Figure 4:**
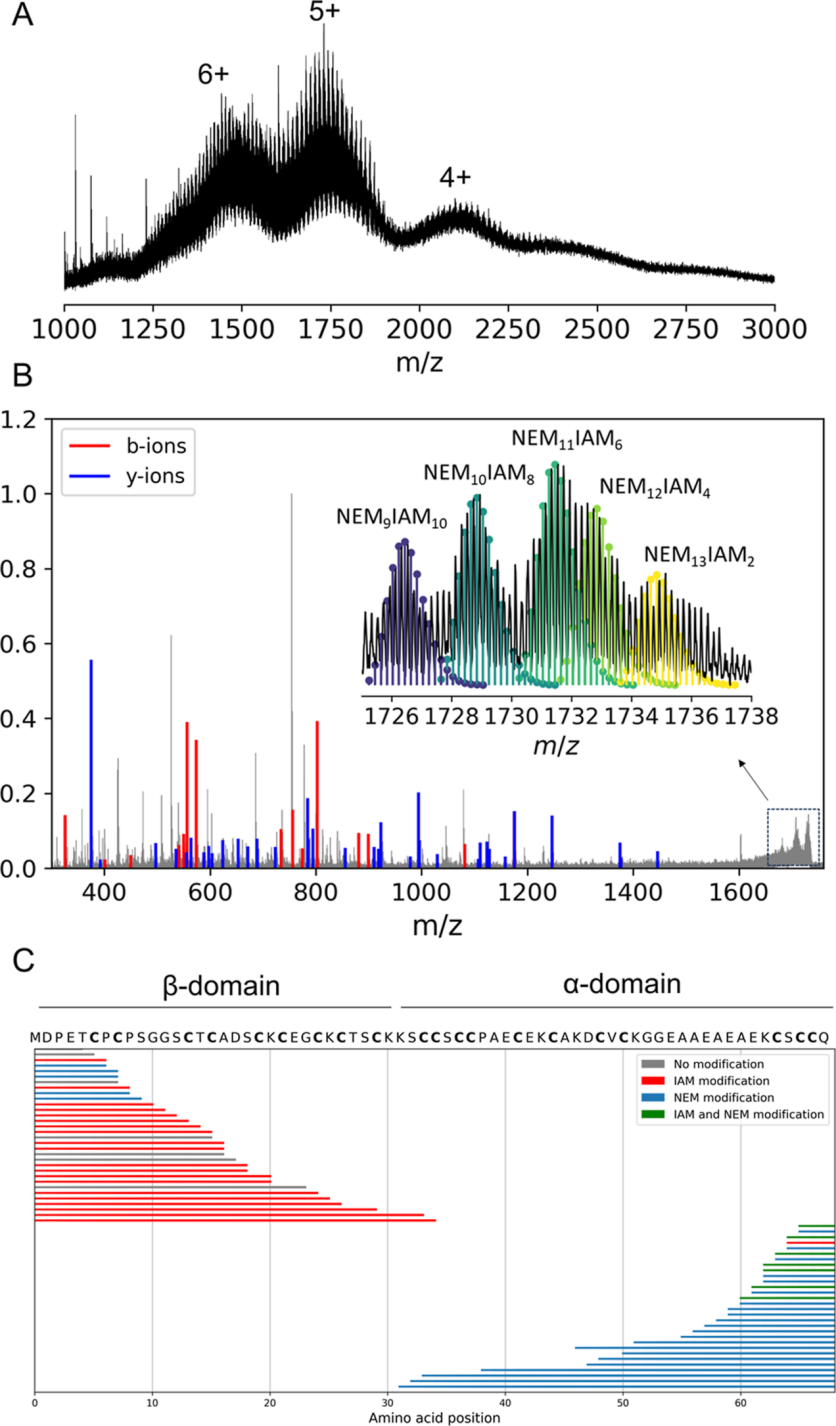
Native mass spectra of NEM- and IAM-Cys labeled residues of the Cu(I)/Zn(II)-MT3 complexes obtained after incubation of Zn_7_MT3 with 4 CuCl_2_ mol eq (A). Native top-down CID MS for quadrupole-selected 1730 *m*/*z* ions (B). CID fragmentation map for the 1730 *m*/*z* ions (C).

In order to further strengthen our conclusions, we extended the single to a double labeling approach (Scheme 1). Briefly, in this step, NEM-labeled Cu(I)-MT3_ox_ complexes were incubated with 100 mM IAM to react with Cys-Cu(I) binding residues. This process involved IAM alkylating Cys residues, resulting in subsequent Cu(I) dissociation. The recorded native mass spectrum shows a vast number of partially overlapping signals originating from multiple IAM stoichiometries for a particular NEM-MT3 complex (Figure 4A). For instance, NEM_12_IAM_3–7_MT3_ox_ or NEM_11_IAM_1–7_MT3_ox_ products were annotated (Figure 4B). We observed a repetitive pattern where three complexes could be annotated within small *m*/*z* windows, following this rule: NEM_*x*_IAM_*y*_MT3_ox_, NEM_*x*+1_IAM_*y*–2_MT3_ox_, and NEM_*x*+2_IAM_*y*–4_MT3_ox_, where *x* and *y* are the NEM and IAM stoichiometry (Figure S15). For example, in the most intense 1730 *m*/*z* ion region, we annotated three complexes: NEM_11_IAM_6_MT3_ox_, NEM_12_IAM_4_MT3_ox_, and NEM_13_IAM_2_MT3_ox_. To localize which Cys residues were modified with IAM and therefore infer where Cu(I) ions were previously bound in the Cu(I)_4_NEM_*x*_MT3_ox_ complex, we selected several *m*/*z* windows and employed top-down MS (Scheme 1). We observed that the fragmentation spectra of these complexes shared most of the ions (approximately 90%), and their intensities showed a high correlation (*R* = 0.97–0.99) (Figure S16). The CID fragmentation map of the 1730 *m*/*z* ions, comprising the above-mentioned complexes, revealed the presence of multiple *b*-fragment ions labeled with varying extents of IAM modifications (Figure 4C).

As in the case of the single NEM labeling approach, the bottom-up MS results were ambiguous as many Cys residues were found to be labeled by both IAM and NEM due to the sample heterogeneity (Figure S17). In this case, the top-down MS approach proves to be beneficial as the dissociation of metals and the alkylation of Cys residues induce protein unfolding, resulting in enhanced fragmentation and achieving high sequence coverage with overlapping *b*- and *y*-fragment ions.

In the previous experiment (Figure 3), the β-domain was metal-protected by the Cu(I)-thiolate cluster against CID fragmentation, and two *b*-type ions corresponding to two proteoforms were identified. We link these findings to our native MS data, revealing the existence of a proteoform with one disulfide and a second proteoform with two disulfides. Here, employing a double labeling approach, we observed the appearance of multiple Cys-IAM labeled *b*-fragment ions covering the β–domain. These findings clearly indicate that Cu(I) is bound to Cys residues in the β–domain of the Cu_4_NEM_*x*_MT3_ox_ complexes. The CID fragmentation map also indicates the existence of two proteoforms: one with a single disulfide and a second one with two disulfides, involving Cys5 and Cys7.

### Application to Rabbit Liver MT2

**Figure 5:**
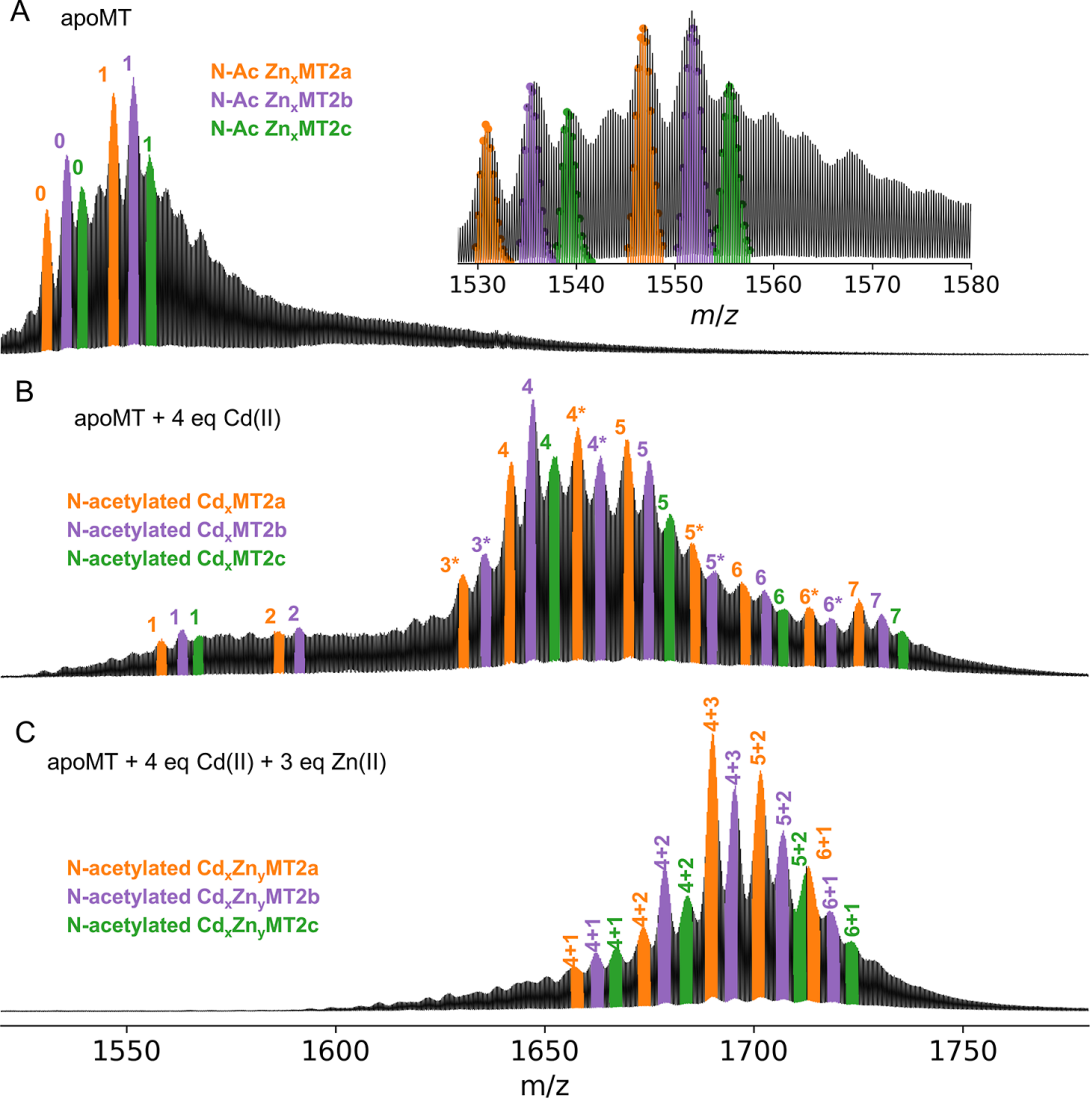
Native mass spectra of rabbit apo-MT (A), incubated with 4 Cd(II) mol eq (B), and after addition of 3 Zn(II) mol eq (C). The *m*/*z* region corresponds to 4+ ions. For the full spectra, see Figure S18. The metalloforms were assigned based on isotopic distribution fitting (see the inset in (A) and Figure S1).

**Figure 6:**
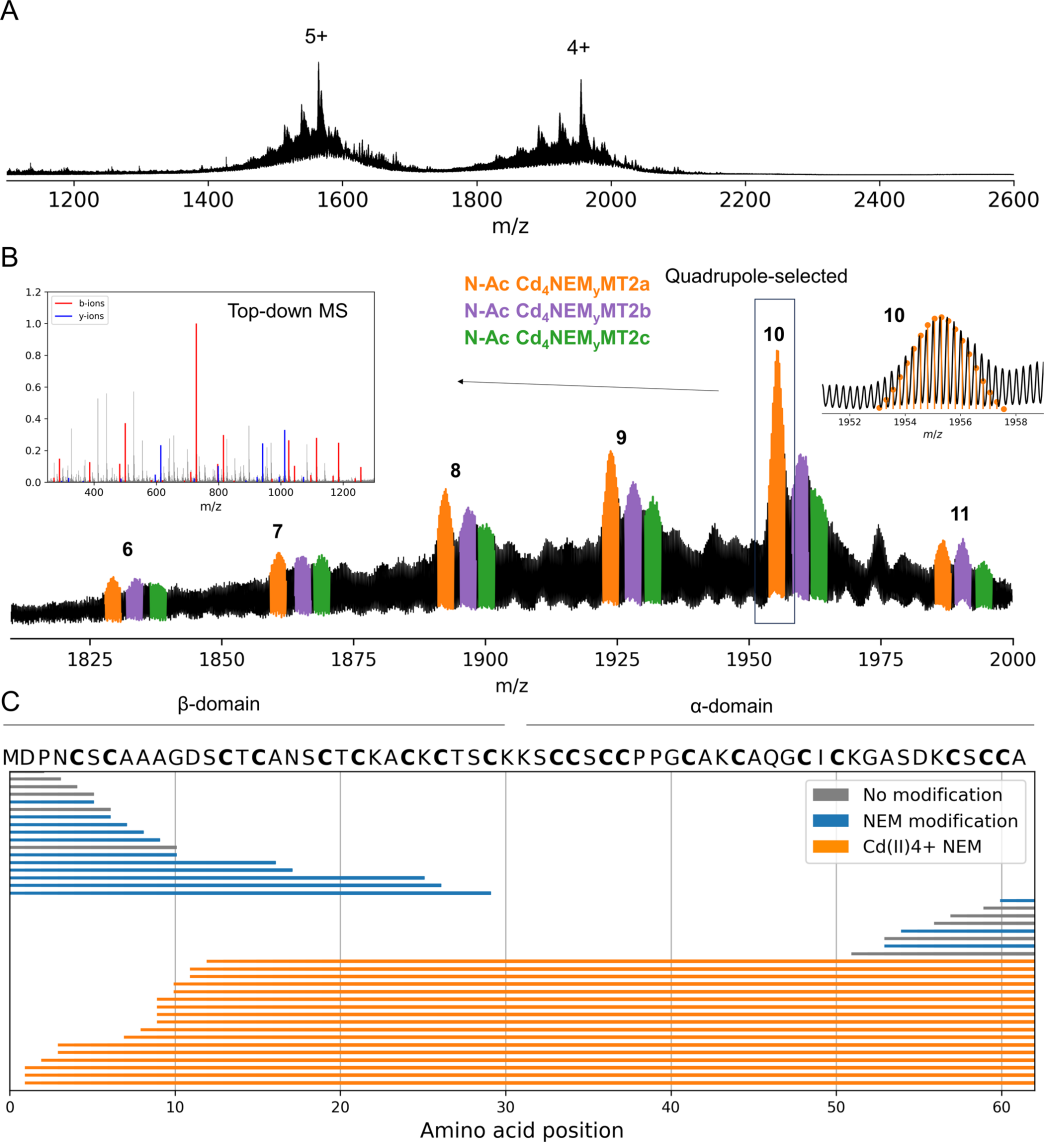
Native mass spectra of rabbit apo-MT after addition of 4 Cd(II) and 3 Zn(II) mol eq. and incubated with 50 mM NEM (A). Zoom in the 4+ ions showing the multiple iso- and proteoforms that differ in the NEM moieties content. These species were isotopically resolved as shown in inset B (B). The N-Ac Cd_4_NEM_10_MT2a 4+ ions were quadrupole-selected and subjected to top-down CID MS. CID fragmentation map for the Cd_4_NEM_10_MT2a 4+ ions (C).

Here, we sought to demonstrate the applicability of the proposed methodology to a mixture of metallothionein isoforms obtained from rabbit liver. The native mass spectra shows two metalloforms for each MT isoform: N-acetylated (N-Ac) MT2a, MT2b, and MT2c isoforms in their apo-form and with one Zn(II) ion bound (Figures 5A and S18A). Purified rabbit liver metallothionein has been found natively binding Zn(II) and Cd(II) ions. Therefore, we prepared these metalloforms in vitro by first adding 4 mol equiv of Cd(II) resulting in seven metalloforms (Figure 5B). This was followed by adding 3 mol equiv of Zn(II), which led to the formation of five metalloforms (Figures 5C and S18B,C). We observed multiple mixed Zn(II)/Cd(II) stoichiometries for each isoform with a maximum of 7 metal ion bound to one protein (Figure 5C). Incubation of that sample with 50 mM NEM leads to N-Ac Cd_4_NEM_6–11_MT2 proteoforms (Figure 6A,B).

The N-Ac Cd_4_NEM_10_MT2 is the most dominant form, revealing that 10 Cys residues are weakly bound to three Zn(II). Considering MT2 has 20 Cys residues, the remaining 10 Cys residues are coordinating 4 Cd(II) ions. Performing a top-down MS shows that the β-domain is mostly modified by NEM, and Cd(II) ions are bound in the α-domain (Figure 6C). The data also show that the C-terminal Cys residues interact weakly with metal ions and can be modified by NEM. Next, we sought to confirm this by the double labeling approach presented above. The intact mass spectra display multiple 4+ and 5+ ions isotopically resolved that can be assigned to two peak series: N-Ac NEM_*x*_IAM_*y*–2_MT2a/b/c and N-Ac NEM_*x*+1_IAM_*y*–4_MT2a/b/c peak series (Figure S19A,B). Importantly, this experiment denotes that IAM reacts with N-Ac Cd_4_NEM_6–11_MT2a/b/c and alkylates the Cys residues that had bound the Cd(II) ions. Top-down MS mapped those IAM moieties bound in the α-domain (Figure S19C).

## Conclusions

The simultaneous mapping of different metal ions bound to cysteine residues presents a significant challenge. In this study, we developed a differential labeling strategy to map Cu(I)/Zn(II) and Cd(II)/Zn(II) metal binding sites within proteins. To accomplish this, we utilized NEM and IAM as Cys labeling probes and Cu(I)/Zn(II) metallothionein-3 as a model protein. Therefore, the methodology presented is limited to map metal-binding Cys residues. Metallothionein-3 plays a crucial role regulating copper and zinc levels in the CNS.^4,42^ Previous studies have shown that MT3 can be isolated from mammalian brains in the form of an air-stable complex known as Cu(I)_4_Zn(II)_3–4_MT3_ox_.^37,38^ A wealth of in vitro studies has demonstrated the feasibility of generating the heterogeneous metalloprotein, Cu(I)/Zn(II) MT3, through the reaction between Zn_7_MT3_red_ and Cu(II).^43−46^ This reaction has been consistently observed to facilitate the binding and subsequent reduction of Cu(II) to Cu(I), leading to the dissociation of Zn(II) ions and the formation of two intramolecular disulfide bonds.

Through our investigations, we prepared a heterogeneous Cu(I)_4_Zn(II)_4_MT3_ox_ complex, incubated it with increasing concentrations of NEM and IAM, and monitored the reactions using native MS. Our findings demonstrate that NEM undergoes cooperative nucleophilic addition with Cys residues, leading to the dissociation of all Zn(II) ions. In contrast, due to the higher affinity of Cu(I) for Cys compared to Zn(II), Cu(I) remains stably bound to the protein. These results emphasize the remarkable capability of NEM to generate homogeneous Cu(I)_*x*_NEM_*y*_MT3_ox_ complexes and illustrate that, under carefully controlled conditions, NEM selectively dissociates all Zn(II) ions while preserving the binding of Cu(I). However, the use of a chemical reaction also introduces a level of complexity, as the alkylation process follows a statistical distribution. For instance, NEM labeling dissociates all four Zn(II) ions coordinated by 11 Cys residues. However, instead of observing a single peak with 11 Cys-NEM labeled residues, we find a statistical distribution ranging from 2 to 13 Cys-NEM modifications.

Determining the precise stoichiometries of alkylator and metal proteins based on only mass information can be challenging due to the presence of isobaric species.^52^ However, our study has demonstrated that through CID experiments, we overcame this challenge and determined the exact stoichiometry between Cu(I) ions and NEM moieties in MT3_ox_ complexes. The CID spectra obtained contained an *m*/*z* region where ions corresponding to the precursor and the precursor with the loss of certain metal ions were observed. This enabled us to directly obtain information regarding the number and type of metal ions bound to the system. In addition, we observed that the Cu(I)_4_NEM_*y*_MT3_ox_ complexes exhibited greater gas-phase stability than NEM_*y*_MT3_ox_, providing further evidence for the stabilizing role of Cu(I) in the protein.

Subsequently, we employed both bottom-up and top-down MS to precisely identify the sites of Cys-NEM modifications in the Cu(I)_4_NEM_*y*_MT3_ox_ complexes, allowing us to deduce the specific locations where Zn(II) ions were originally bound within the Cu(I)_4_Zn(II)_4_MT3_ox_ complex. Our observations from the peptide-centric data yielded ambiguous results, as all Cys residues within the protein were found to be labeled with NEM modifications. It can be attributed to the presence of a portion of NEM_16–20_MT3_ox_^5+^ ions alongside the formation of homogeneous Cu(I)_4_NEM_*x*_MT3_ox_^5+^ complexes (*x* = 8–12), as NEM reacts with the protein. The implementation of a bottom-up MS approach in this strategy becomes infeasible due to its larger dynamic range compared to native MS.

Alternatively, through native top-down MS, we successfully mapped all the residues labeled with Cys-NEM modifications. Our findings revealed that the α-domain of the protein was indeed labeled with NEM, while the β-domain demonstrated binding of four Cu(I) ions. However, it is important to note that we did not observe fragmentation within the protein region where Cu(I) is bound.

In order to get insights into the Cu(I) binding sites, we introduced a second labeling step in our strategy using IAM. Following the initial labeling with NEM, NEM-labeled Cu(I)-MT3_ox_ complexes were incubated with IAM. IAM reacted with the Cys residues involved in Cu(I) coordination, resulting in their alkylation and subsequent dissociation of Cu(I) from the protein. The native mass spectrum we recorded displayed numerous partially overlapping signals originating from multiple IAM stoichiometries for a specific NEM-MT3_ox_ complex. Similar to previous findings, the bottom-up MS results were inconclusive due to the sample heterogeneity, but the top-down MS approach yielded valuable information. Examining the CID fragmentation map of the most intense *m*/*z* window region (1730 *m*/*z* ions), we annotated multiple *b*-fragment ions labeled with varying extents of IAM modifications. These findings provide clear evidence that Cu(I) is bound to Cys residues, specifically within the β-domain of the Cu_4_NEM_*x*_MT3 complexes. Finally, we have applied the proposed methodology to infer the location of Cd(II) and Zn(II) ions in the rabbit liver metallothionein fraction, which contains multiple MT isoforms and post-translational modifications.

To conclude, the described approach holds promise for further exploration of the Cu(I)/Zn(II) and Cd(II)/Zn(II) metalloforms’ diversity within other proteins and expand its spectrum to other metal ions.

## Data Availability

The mass spectrometry data have been deposited to Figshare repository (10.6084/m9.figshare.25904197).

## Supporting Information Available

Materials and methods; expression and purification of metallothionein-3; UV–vis spectroscopy; MS; native top-down CID MS; single and double Cys labeling preparation; rabbit liver metallothionein preparation; computational workflow for isotopic fitting; absorption spectra of Zn_7_MT3 upon reaction with CuCl_2_; native mass spectra of Zn_7_MT3 incubated with CuCl_2_ and IAM; schematic representation of the NEM labeling reaction; native mass spectra of Zn_7_MT3 incubated with CuCl_2_ and NEM; native top-down CID MS spectrum for Cu(I)_4_Zn(II)_4_MT3_ox_^5+^ ions; CID mass spectra for Figure 2; native top-down CID MS spectrum for Cu(I)_5_NEM_8_MT3_ox_^5+^ and Cu(I)_5_NEM_10_MT3_ox_^5+^; CID MS spectrum for different Cu(I) protein complexes; SY plots; native top-down for different Cu(I) protein complexes; native top-down for Cu(I)_4_NEM_8_MT3_ox_^5+^; native top-down for Cu(I)_4_NEM_8_MT3_ox_^5+^; BU coverage map and peptide-mass fingerprint of Zn_7_MT3 incubated with CuCl_2_ and NEM; native MS of NEM and IAM-Cys labeled of Cu(I)/Zn(II)-MT3 complexes; analysis top-down MS for NEM and IAM-Cys labeled of Cu(I)/Zn(II)-MT3 complexes; BU coverage map and peptide-mass fingerprint of Zn_7_MT3 incubated with CuCl_2_, IAM, and NEM; native MS of rabbit apo-metallothionein with Cd(II), Zn(II), and NEM; native MS of rabbit MT incubated with Cd(II), Zn(II), NEM, and IAM, and top-down MS experiments; and references (PDF)
