# Supplementary material for: Combining Native Mass Spectrometry and Proteomics to Differentiate and Map the Metalloform Landscape in Metallothioneins
Source: J Proteome Res. 2024 Jul 12;23(8):3626–37. doi: 10.1021/acs.jproteome.4c00271 (PMC11301679; doi:10.1021/acs.jproteome.4c00271)
Supplement: Supplementary file 1 — pr4c00271_si_001.pdf [file pr4c00271_si_001.pdf]

Supporting Information

Combining native mass spectrometry and proteomics to differentiate and map the metalloform landscape in metallothioneins

Manuel David Peris-Díaz[12]*†, Alicja Orzeł[1], Sylwia Wu[1], Karolina Mosna[1], Perdita E. Barran[2], Artur Krężel[1]*

[1]Department of Chemical Biology, Faculty of Biotechnology, University of Wrocław, F. Joliot-Curie 14a, 50-383 Wrocław, Poland. [2]Michael Barber Centre for Collaborative Mass Spectrometry, Manchester Institute of Biotechnology, 131 Princess Street, Manchester, M1 7DN, United Kingdom.

TABLE OF CONTENTS

Materials. 2

Expression and purification of metallothionein-3 2

UV-vis spectroscopy....3

Mass spectrometry. 3

Native top-down CID mass spectrometry....4

Single and double Cys labeling preparation. 4

Rabbit liver metallothionein 5

Figure S1. Computational workflow for isotopic fitting 6

Figure S2. Absorption spectra of Zn_7MT3 upon reaction with $CuCl_2$....7

Figure S3. Native mass spectra of Zn_7MT3 incubated with $CuCl_2$ and IAM 8

Figure S4. Schematic representation of the NEM labeling reaction 9

Figure S5. Native mass spectra of Zn_7MT3 incubated with $CuCl_2$ and NEM....10

Figure S6. Native top-down CID MS spectrum for $Cu(I)_4Zn(II)_4MT3_{ox}^{5+}$ ions. 11

Figure S7. CID mass spectra for Figure 2 12

Figure S8. Native top-down CID MS spectrum for $Cu(I)_5NEM_8MT3_{ox}^{5+}$ and $Cu(I)_5NEM_{10}MT3_{ox}^{5+}$...13

Figure S9. CID MS spectrum for different Cu(I) protein complexes....14

Figure S10. Survival yield plots 15

Figure S11. Native top-down for different Cu(I) protein complexes 16

Figure S12. Native top-down for $Cu(I)_4NEM_8MT3_{ox}^{5+}$....17

Figure S13. Top-down ETD spectra for $Cu(I)_4NEM_8MT3_{ox}^{5+}$....17

Figure S14. BU coverage map and peptide-mass fingerprint of Zn_7MT3 incubated with $CuCl_2$ and NEM 18

Figure S15. Native MS of NEM and IAM-Cys labeled of Cu(I)/Zn(II)-MT3 complexes 19

Figure S16. Analysis top-down MS for NEM and IAM-Cys labeled of Cu(I)/Zn(II)-MT3 complexes 20

Figure S17. BU coverage map and peptide-mass fingerprint of Zn_7MT3 incubated with $CuCl_2$, IAM and NEM....21
Figure S18. Native MS of rabbit apo-metallothionein with Cd(II), Zn(II) and NEM....22
Figure S19. Native MS of rabbit MT incubated with Cd(II), Zn(II), NEM and IAM, and top-down MS experiments....23
REFERENCES....24

EXPERIMENTAL SECTION

Materials. The reagents used in this study were purchased from Sigma-Aldrich, Merck, Acros Organics, Roth, BioShop, VWR International (Avantor), and Iris-Biotech GmbH. The following reagents: $ZnSO_4 \cdot 7H_2O$, 4-(2-pyridylazo)resorcinol (PAR), $(NH_4)_2CO_3$, tris(hydroxymethyl)aminomethane (Tris base) and 4-(2-hydroxyethyl)-1 piperazineethanesulfonic acid (HEPES), mass spectrometry grade methanol, tris(2carboxyethyl)phosphine hydrochloride (TCEP), ammonium acetate (AmAc), ammonium bicarbonate, ethylenediamine-tetraacetic acid (EDTA), and mass spectrometry grade acetonitrile (ACN) were purchased from Sigma-Aldrich. Resin Chelex 100 was acquired from Bio-Rad and 98% hydrochloric acid (HCl) was purchased from VWR Chemicals. DL-dithiothreitol (DTT) was purchased from Iris Biotech GmbH. Tryptone, LB broth, yeast extract, isopropyl-β-D-1-thiogalactopyranoside (IPTG), and SDS were from Lab Empire, NaCl, NaOH, glycerol, $KH_2PO_4 \cdot H_2O$, K_2HPO_4 from POCH (Gliwice Poland), pTYB21 vector and chitin resin were from New England BioLabs, and 5,5′-dithiobis-(2-nitrobenzoic acid) (DTNB) from TCI Europe.

Expression and purification of metallothionein-3. The expression vector (Addgene plasmid ID 105710) containing metallothionein was transformed into BL21(DE3) *E. coli* cells, which were then grown in a culture medium (1.1% tryptone, 2.2% yeast extract, 0.45% glycerol, 1.3% K_2HPO_4, 0.38% KH_2PO_4) at 37°C until ~0.8 OD_{600}. Protein expression was induced by adding 0.1 mM IPTG along with 0.5 M $ZnSO_4$ to the cells, followed by overnight incubation at 20°C with shaking. Subsequent steps were performed at 4°C. The cells were harvested by centrifugation at 4,000 · *g* for 10 min and then resuspended in 50 mL of cold buffer A (20 mM HEPES, pH 8.0, 500 mM NaCl, 1 mM EDTA, 1 mM TCEP). After sonication for 45 min (using 5 s sonication and 10 s pause cycles), the cell lysate was obtained by centrifugation at 16,000 · *g* for 15 min. The resulting supernatant was loaded onto a chitin resin and incubated overnight

with buffer A (20 ml). Following this, the resin was washed with buffer A (50 ml) and treated with 100 mM DTT for cleavage. The resin was then incubated on a rocking bed at room temperature for 48 h. The eluted solution from the chitin column was concentrated using Amicon Ultra-4 Centrifugal Filter Units with a 3 kDa membrane cut-off (Merck Millipore, USA). Subsequently, the pH of the concentrated solution was lowered to approximately 2.5 by adding 7% HCl, and another centrifugation step was performed. The protein was then purified on a size exclusion chromatography using a HiLoad 16/600 Superdex 75 pg gel filtration column (Cytiva, USA) equilibrated with 10 mM HCl at 1 ml/min flow rate using an ÄKTA Pure system.[1] The identity of the protein in the eluted fractions was confirmed using ESI-MS with a Bruker Maxis Impact (Bruker Daltonik GmbH, Bremen, Germany) calibrated with a commercial ESI-TOF Tuning mix (Sigma-Aldrich). The concentration of thiols was determined spectrophotometrically using a DTNB assay[2], while the Zn(II) binding capacity was confirmed spectrophotometrically by Zn(II) and Cd(II) titrations.[3] After obtaining the purified apoMT3, an 8.5 molar excess of $ZnSO_4$ was added to it in the presence of a nitrogen blanket, and 1 mM TCEP to prevent oxidation. The pH of the solution was adjusted to 8.6 using a 1 M Tris base. The resulting mixture was concentrated using Amicon Ultra-4 Centrifugal Filter Units with a 3 kDa membrane cut-off (Merck Millipore, USA). Subsequently, the concentration samples were subjected to purification on a SEC HiLoad 16/600 Superdex 75 pg gel filtration column (Cytiva, USA) that had been equilibrated with 20 mM Tris-HCl buffer at pH 8.6. The concentrations of thiols and Zn(II) were determined spectrophotometrically using DTNB and PAR assays, respectively.[4]

UV-vis spectroscopy. The spectroscopic titrations were carried out under anaerobic conditions, ensuring the absence of oxygen. All reagents used in the experiment were degassed before being placed in the glove box. The reaction involved 2.5 µM Zn_7MT3_{red} and Cu(II) (provided as $CuCl_2$ in 20 µM HCl) in chelexed borate buffer containing 100 mM NaF at a pH 7.4. The progress of the reaction was monitored by measuring the absorbance in the wavelength range of 210-450 nm. The samples were allow to equilibrate for 2.5 min, which was sufficient to reach a plateau in the kinetic mode specifically at a wavelength of 255 nm.

Mass spectrometry. MS experiments were carried out on a Synapt XS HDMS equipped with nanoelectrospray ionization (Waters Corporation, Manchester, UK). Samples were prepared at 10-20 µM in 200 mM ammonium acetate (AmAc), pH 6.8, and desalted using micro Bio-Spin 6 columns (Bio-Rad). 3-10 µL of sample were loaded into borosilicate glass capillaries (O.D.

1.2 mm, I.D. 0.9 mm, World Precision Instruments, Stevenage, UK) produced in-house using a Flaming/Brown P-1000 micropipette puller (Sutter Instrument Co., Novato, CA, USA) and ions were produced by applying a positive potential of 0.9-1.4 kV via a platinum wire (Goodfellow). All of the experiments were performed in sensitivity mode to maximize ion transmission. Native MS experiments were done by using source conditions (source temperature 30°C, cone voltage 20 V, source offset 1), trap cell collision energy (5 V) as well as bias potentials (trap DC bias 35 V) that prevented metal ion dissociation. The time of flight was calibrated using 2 μg · μL^{-1} NaI made up in 1:1 water:isopropanol. Data were analysed by means MassLynx v4.2 (Waters Corp., UK), and custom scripts in Python 3.5 (available in https://github.com/ManuelPerisDiaz/Cu-Zn-MT3).

Native top-down CID mass spectrometry. Native top-down collision-induced dissociation (CID) mass spectrometry experiments were performed by applying 20-60 V of trap collision energies of quadrupole-selected ions. Argon gas was used as the collision gas during the experiments. Equation 1 was utilized to determine the survival yield of precursor ions:

$$\mathrm{SY} = \frac{I_{\mathrm{P}}}{I_{\mathrm{P}} + \sum I_{\mathrm{F}}} \qquad (1)$$

where I_{P} and I_{F} refers to the intensity of precursor and fragment ions, respectively.[5] Trap voltages ($\mathrm{trap_{CE}}$) were transformed to center-of-mass energies (E_{com}) using eq 2:

$$E_{\mathrm{com}} = \frac{\mathrm{m_g}}{\mathrm{m_g} + \mathrm{m_p}} \cdot \mathrm{trap_{CE}} \cdot z \qquad (2)$$

where $\mathrm{m_g}$, $\mathrm{m_p}$ and z denote the mass gas (N_2) and mass and charge state of precursor ion, respectively. The survival plots, representing the relationship between survival yield (SY) and the center-of-mass snergies (E_{com}), were fitted to a sigmoid function using non-linear least squares implemented in SciPy version 1.10.0.[6] To perform the analysis, the averaged raw mass spectrum was converted to a text file format. Peak picking and deisotopic were carried out using mMass wigh a signal-to-noise (S/N) threshold of 3, an isotope mass tolerance of 0.05 m/z, and an isotope intensity tolerance of 50%.[7] The experimental peak list obtained was then matched to a theoretical list of b/y fragment ions, accounting for neutral losses, within a 10 ppm accuracy threshold. The results were exported as CSV files for further analysis, which was conducted using custom Python 3.5 scripts.

Single and double Cys labeling preparation. To the Zn_7MT3_{red} stock (1 mM), 4 mol. Eq of $CuCl_2$ prepared in a 20 μM HCl solution were added, and the mixture was incubated for 15 min

on ice. The sample was then desalted using 7 kDa cut-off Zeba spin desalting columns (Thermo Fisher Scientific, USA) equilibrated with 200 mM ammonium acetate (pH 6.8). Various amounts of IAM or NEM (10, 25, 50, 75, 100 mM) were added to the desalted sample to reach a final protein concentration of 45 µM. The mixture was then incubated in the dark at 25°C for 10 min. The stocks of IAM/NEM were prepared in 200 mM ammonium acetate (pH 6.8). The sample was then desalted using 7 kDa cut-off Zeba spin desalting columns (Thermo Fisher Scientific, USA) equilibrated with 200 mM ammonium acetate (pH 6.8).

At this stage, the samples underwent different procedures based on the specific experiments:

1. The samples were either diluted to a concentration of 10 µM for native MS or top-down MS experiments.

2. They underwent buffer exchange to 100 mM ammonium bicarbonate for bottom-up MS experiments.

3. They underwent double labeling with IAM: 100 mM IAM (stock prepared at 750 mM) was added to the sample and incubated in the dark at 25 ºC for 30 min. The sample was then desalted using 7 kDa cut-off Zeba spin desalting columns (Thermo Fisher Scientific, USA) equilibrated with 200 mM ammonium acetate (pH 6.8). At this stage, the samples underwent different procedures based on the specific experiments:

3.1 The samples were either diluted to a concentration of 10 µM for native/denaturing MS or top-down MS experiments.

3.2 They underwent buffer exchange to 100 mM ammonium bicarbonate for bottom-up MS experiments.

Rabbit liver metallothionein. Apo-MT2 was prepared from isolated from rabbit liver (Santa Cruz Biotechnology, Inc, USA) by acidification with concentrated HCl in the presence of 1 mM TCEP, to prevent oxidation and desalted on PD SpinTrap™ G-25 column (Cytiva, USA). 30 µM Cd_4Zn_3MT2 complex was prepared from apo-MT2, diluted in 150 mM ammonium acetate (pH 7.4), by adding 4 mol. Eq of $Cd(CH_3COO)_2$ and 3 mol. Eq. of $Zn(CH_3COO)_2$, and the mixture was incubated for 15 min on ice. Then the complex was labeled with 50 mM NEM (stock in 150 mM ammonium acetate (pH 7.4)), incubated in the dark at 25 °C for 15 min and desalted on Zeba Micro Spin Desalting Columns (Thermo Fisher Scientific, USA), equilibrated with 150 mM ammonium acetate (pH 7.4). For double labeling, 100 mM IAM was added to the sample and incubated in the dark at 25 °C for 30 min. The sample was desalted using 7 kDa

cut-off Zeba Micro Spin Desalting Columns, equilibrated with 150 mM ammonium acetate (pH 7.4). After each step, a portion of sample underwent for specific experiments.

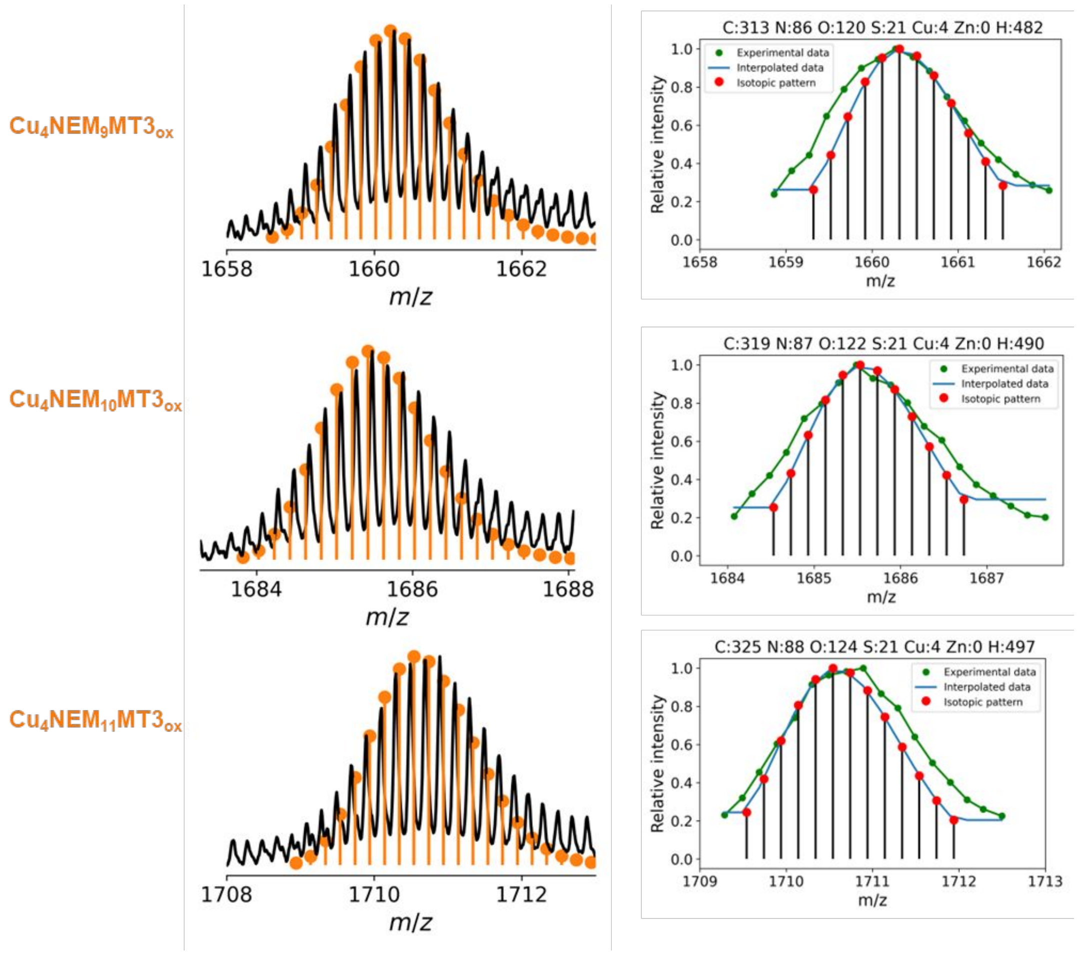

Figure S1. Computational workflow involves generating multiple isotopic patterns, fitting them to the experimental data, and scoring the results. The experimental data included detected peaks for $Cu_4NEM_{9\text{-}11}MT3_{ox}$ proteins, along with simulations of theoretical isotopic patterns with variable proton number ("H"). The filtered theoretical isotopic pattern was then filtered based on intensity threshold (20%), followed by interpolation onto the m/z values of the experimental data (blue line). The molecular formula that provided the best R2 and the lowest chi-square among all the tested candidates was selected.

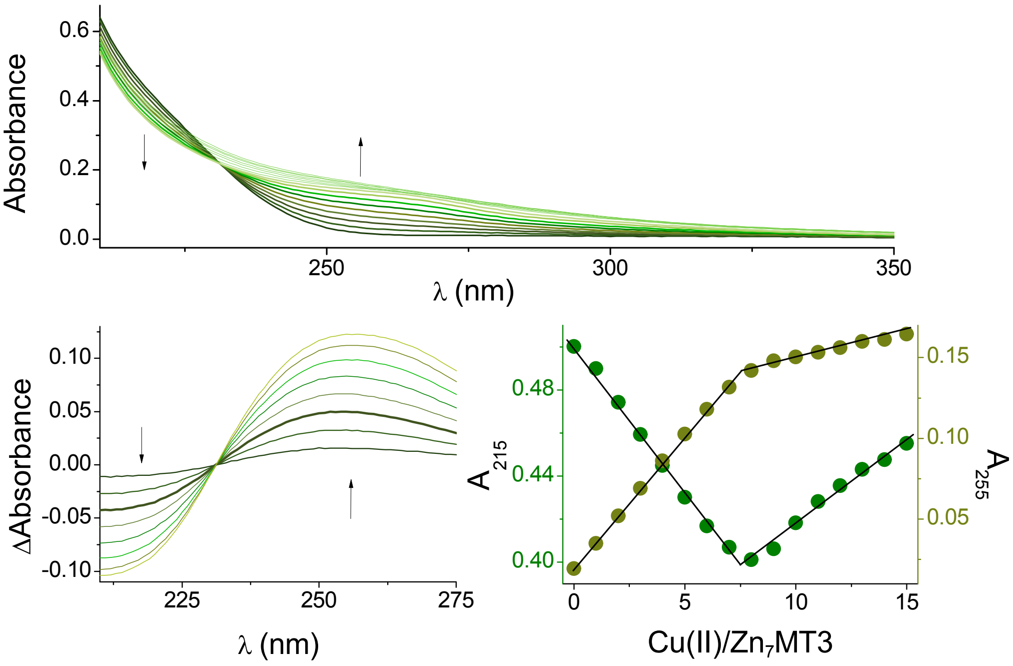

Figure S2. Absorption spectra of 2.5 μM Zn_7MT3_{red} in 100 mM borate buffer (100 mM NaF, pH 7.4) upon reaction with 0–15 molar mol. Eq. of $CuCl_2$ (A). Differential absorption spectra at wavelengths ranging 210-300 nm calculated by subtracting the Zn_7MT3_{red} spectrum from each spectrum after the addition of $CuCl_2$, in the range of 1–8 $CuCl_2$ equivalents (B). Changes at 215 nm and 255 nm resulting from the addition of 0–15 $CuCl_2$ mol Eq (C).

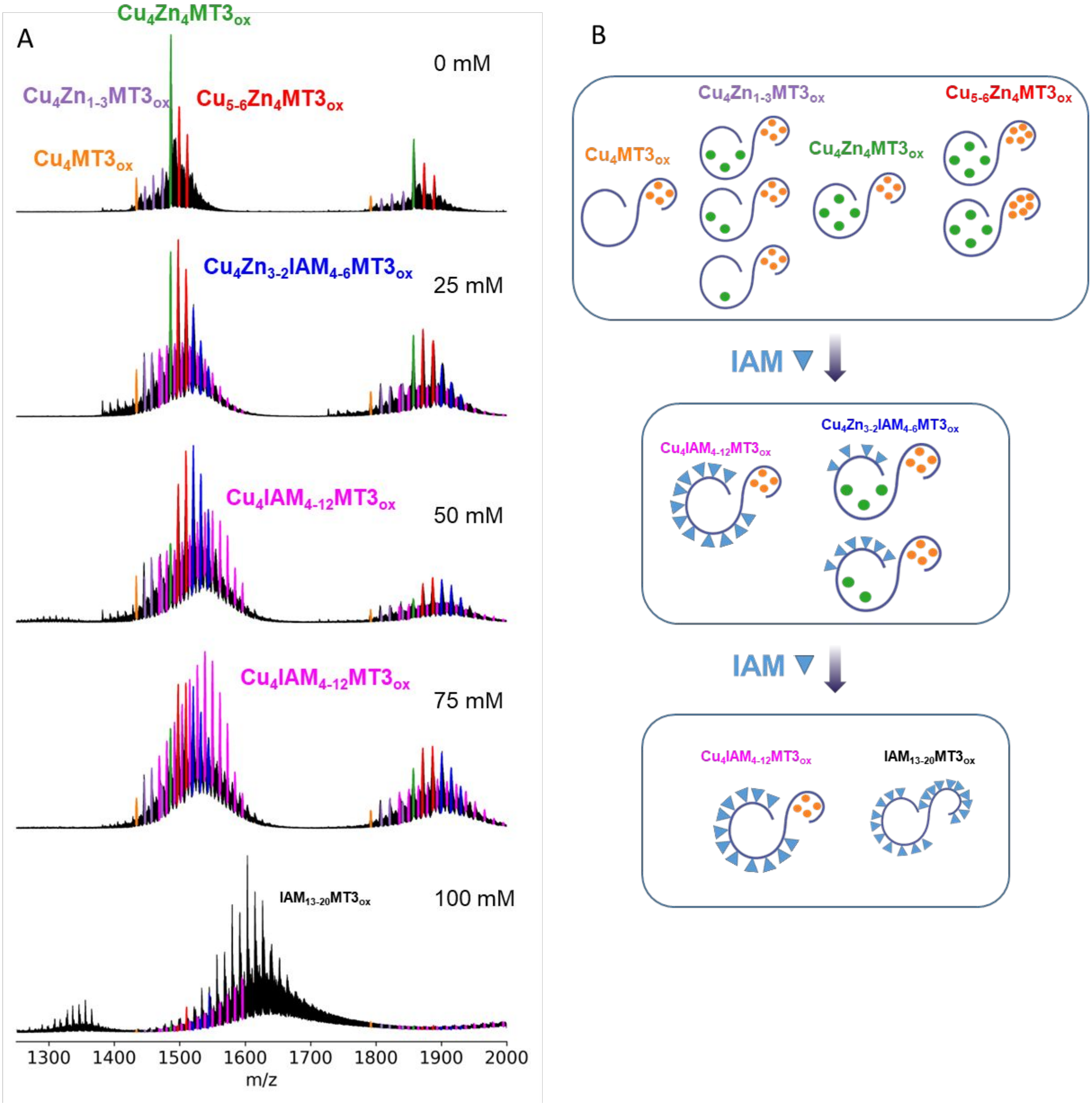

Figure S3. Native mass spectra of Zn_7MT3 incubated with 4 $CuCl_2$ and the resulting products after incubation with different amount of IAM (A). The peaks are coloured according to the Cu(I)/Zn(II) stoichiometry and represent different IAM stoichiometries. The proteins (10 µM) were sprayed in 200 mM ammonium acetate (pH 6.8). The subscript "ox" refers to oxidized (2 intramolecular disulfides) MT3 proteins. A schematic representation of the IAM labeling reaction (B).

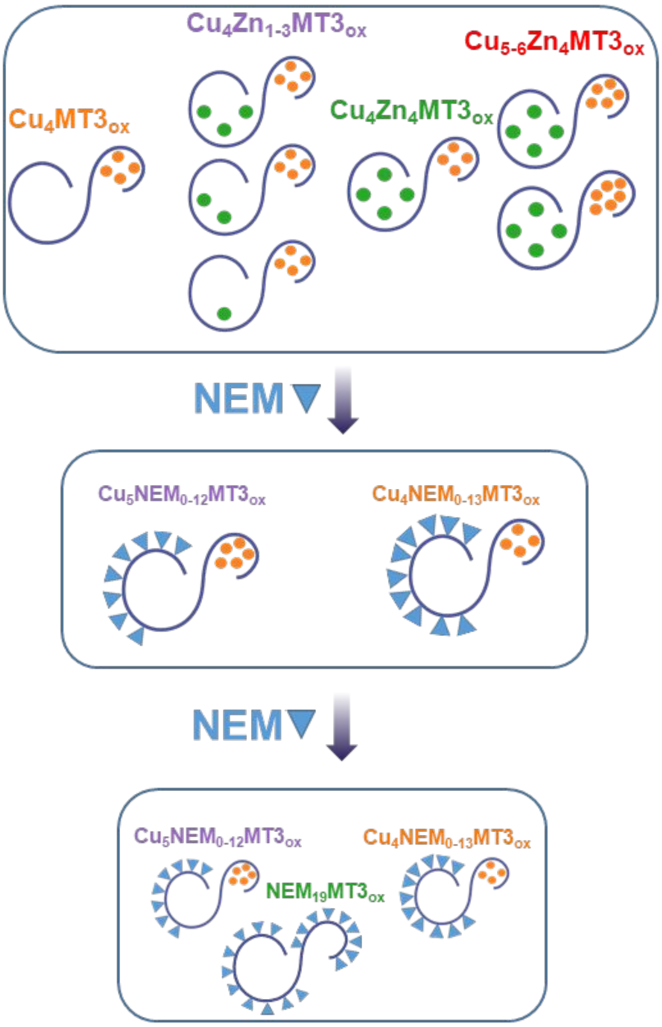

Figure S4. A schematic representation of the NEM labeling reaction for the native MS experiments shown in Figure 1.

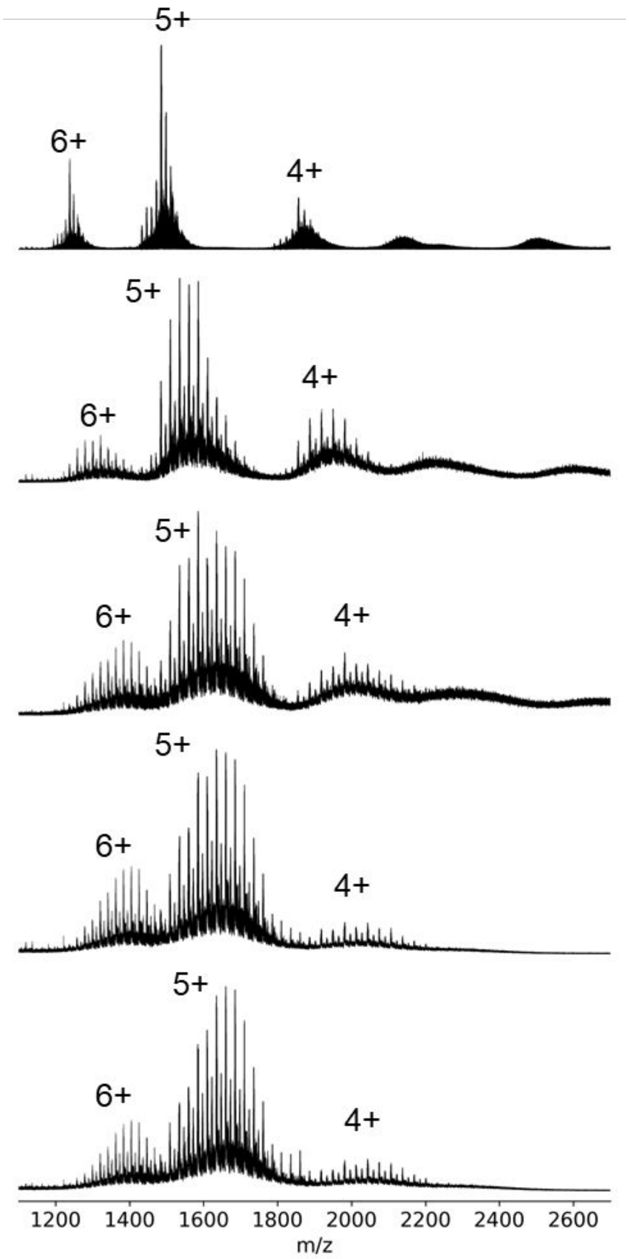

Figure S5. Native mass spectra of the Cu(I)/Zn(II)-$MT3_{ox}$ complexes obtained after incubation of Zn_7MT3_{red} with 4 $CuCl_2$ mol Eq and at increasing concentration of *N*-ethylmaleimide (NEM).

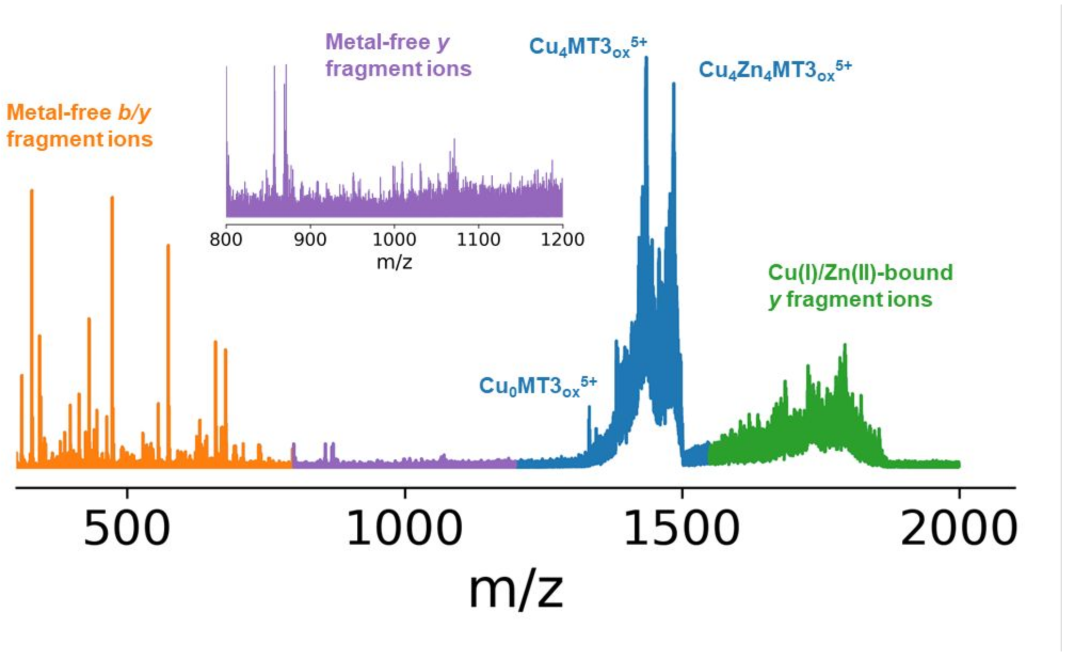

Figure S6. Native top-down CID MS spectrum for quadrupole-selected $Cu(I)_4Zn(II)_4MT3_{ox}^{5+}$ ions. The proteins (10 μM) were sprayed in 200 mM ammonium acetate (pH 6.8) and activated in the trap collision cell.

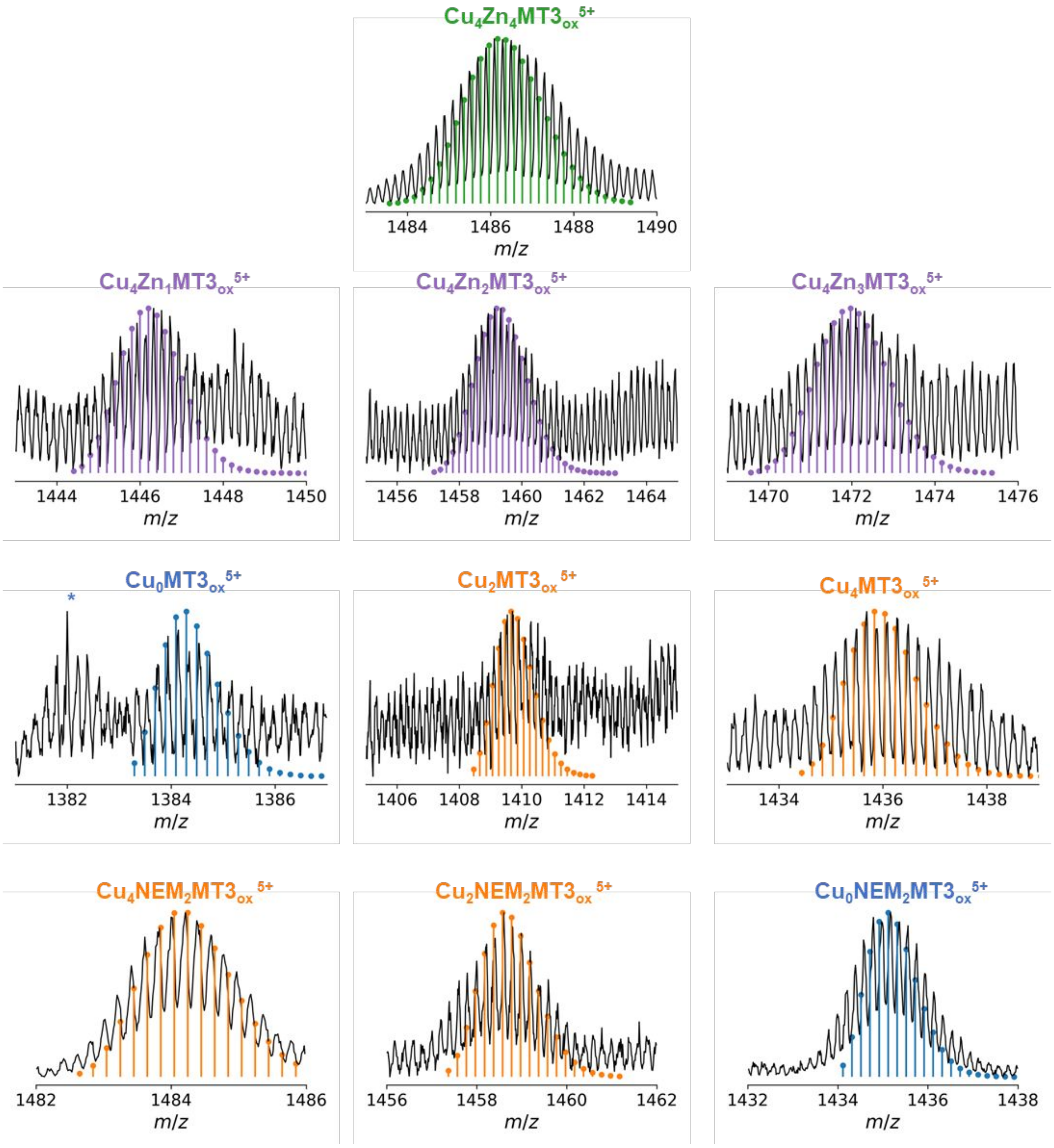

Figure S7. Experimental signals for the collision-induced dissociation (CID) experiments in Figure 2. Data were fitted to isotopic distributions and assigned them followed the methodology in Figure S1. * indicates oxidation product.

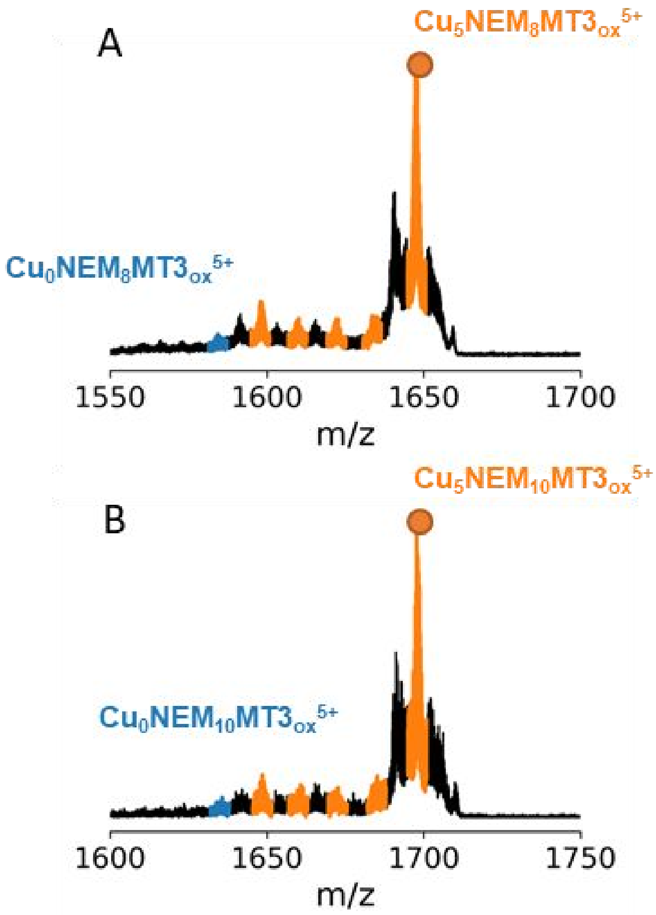

Figure S8. Native top-down CID MS spectrum for quadrupole-selected $Cu(I)_5NEM_8MT3_{ox}^{5+}$ (A) and $Cu(I)_5NEM_{10}MT3_{ox}^{5+}$ (B) ions. The proteins (10 μM) were sprayed in 200 mM ammonium acetate (pH 6.8) and activated in the trap collision cell.

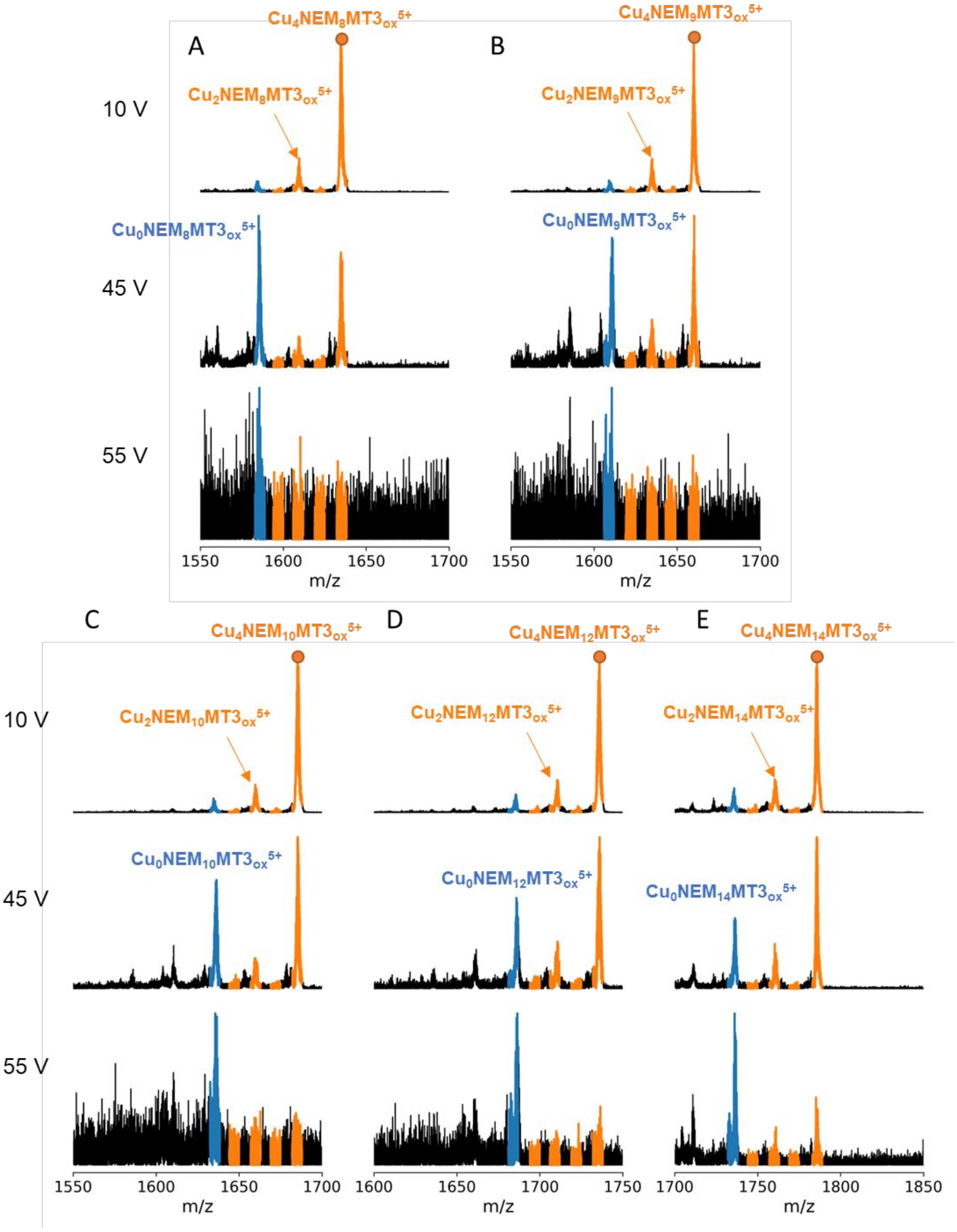

Figure S9. CID experiments for quadrupole-selected $Cu(I)_4NEM_8MT3_{ox}^{5+}$ (A), $Cu(I)_4NEM_9MT3_{ox}^{5+}$ (B), $Cu(I)_4NEM_{10}MT3_{ox}^{5+}$ (C), $Cu(I)_4NEM_{12}MT3_{ox}^{5+}$ (D), $Cu(I)_4NEM_{14}MT3_{ox}^{5+}$ (E). The proteins (10 μM) were sprayed in 200 mM ammonium acetate (pH 6.8) and activated in the trap collision cell at 10, 45 and 55 V.

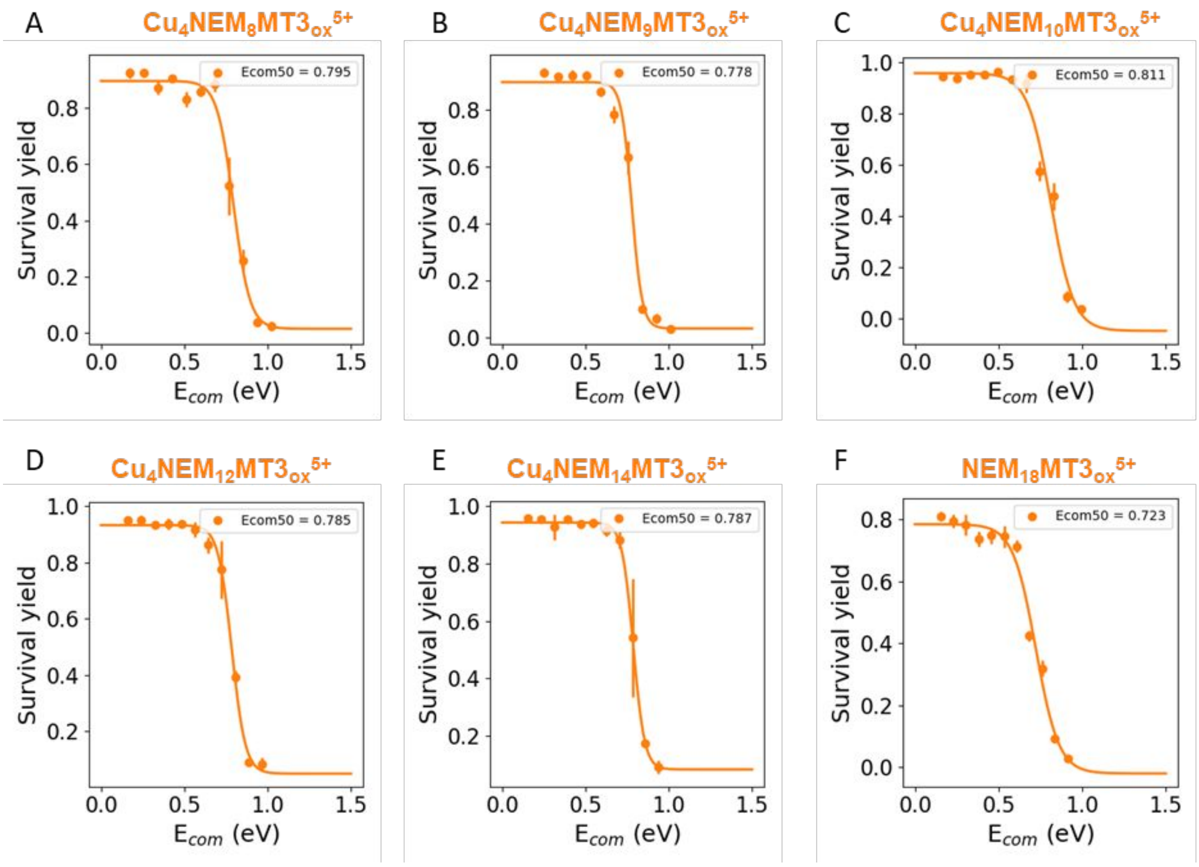

Figure S10. Survival yield plots for quadrupole-selected Cu(I)$_4$NEM$_8$MT3$_{ox}^{5+}$ (A), Cu(I)$_4$NEM$_9$MT3$_{ox}^{5+}$ (B), Cu(I)$_4$NEM$_{10}$MT3$_{ox}^{5+}$ (C), Cu(I)$_4$NEM$_{12}$MT3$_{ox}^{5+}$ (D), Cu(I)$_4$NEM$_{14}$MT3$_{ox}^{5+}$ (E) and NEM$_{18}$MT3$_{ox}^{5+}$ (F) ions . The proteins (10 µM) were sprayed in 200 mM ammonium acetate (pH 6.8) and activated in the trap collision cell at increasing energies as describe above.

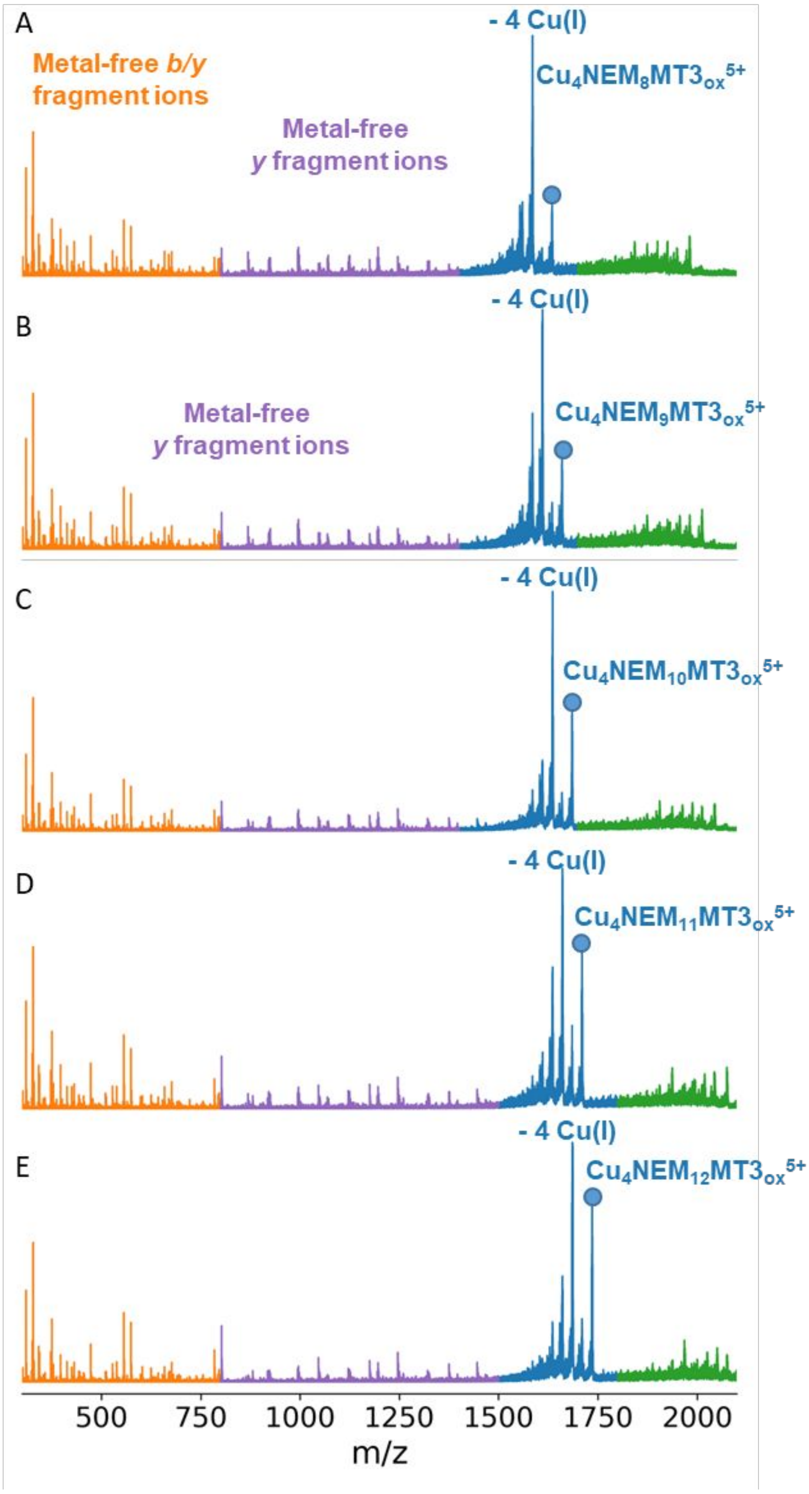

Figure S11. Native top-down CID MS spectrum for quadrupole-selected $Cu(I)_4NEM_8MT3_{ox}^{5+}$ (A), $Cu(I)_4NEM_9MT3_{ox}^{5+}$ (B), $Cu(I)_4NEM_{10}MT3_{ox}^{5+}$ (C), $Cu(I)_4NEM_{12}MT3_{ox}^{5+}$ (D), $Cu(I)_4NEM_{14}MT3_{ox}^{5+}$ (E). The proteins (10 μM) were sprayed in 200 mM ammonium acetate (pH 6.8) and activated in the trap collision cell at 10, 45 and 55 V.

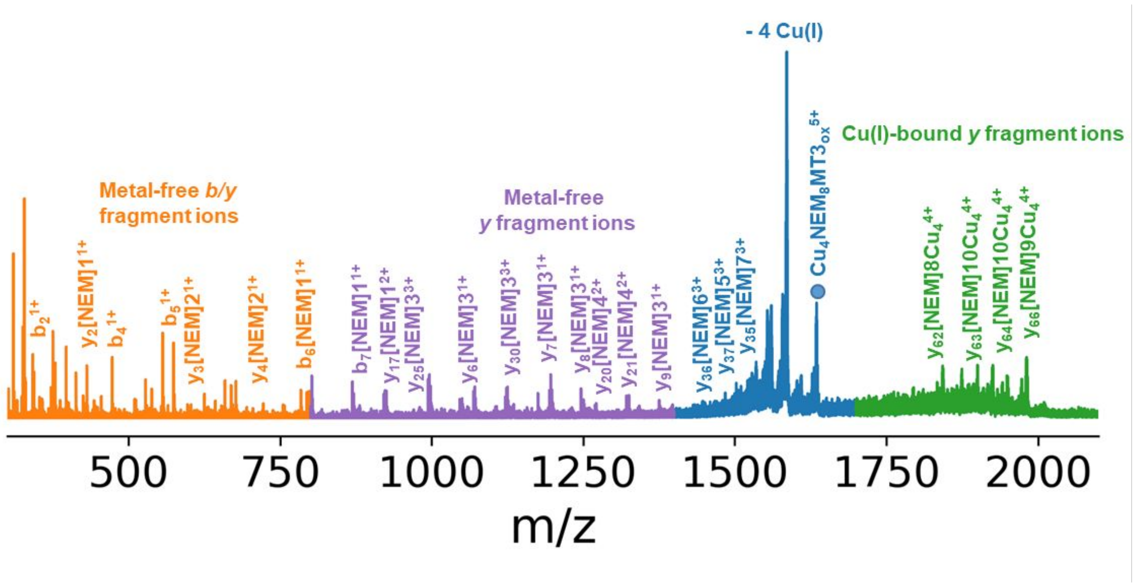

Figure S12. Native top-down CID MS spectrum for quadrupole-selected Cu(I)$_4$NEM$_8$MT3$_{ox}$$^{5+}$.

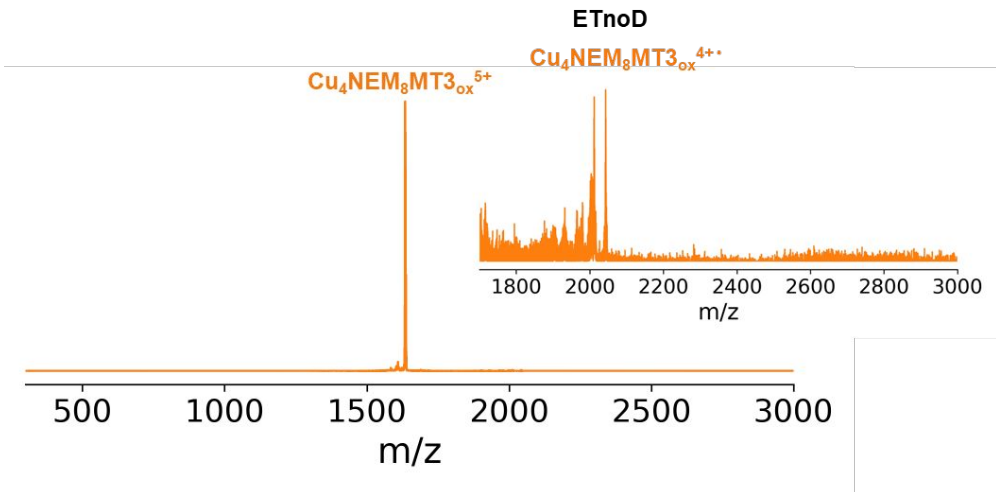

Figure S13. Top-down electron transfer dissociation (ETD) MS for mass-selected Cu(I)$_4$NEM$_8$MT3$_{ox}$$^{5+}$ ions. The protein (10 μM) was sprayed in 200 mM ammonium acetate (pH 6.8).

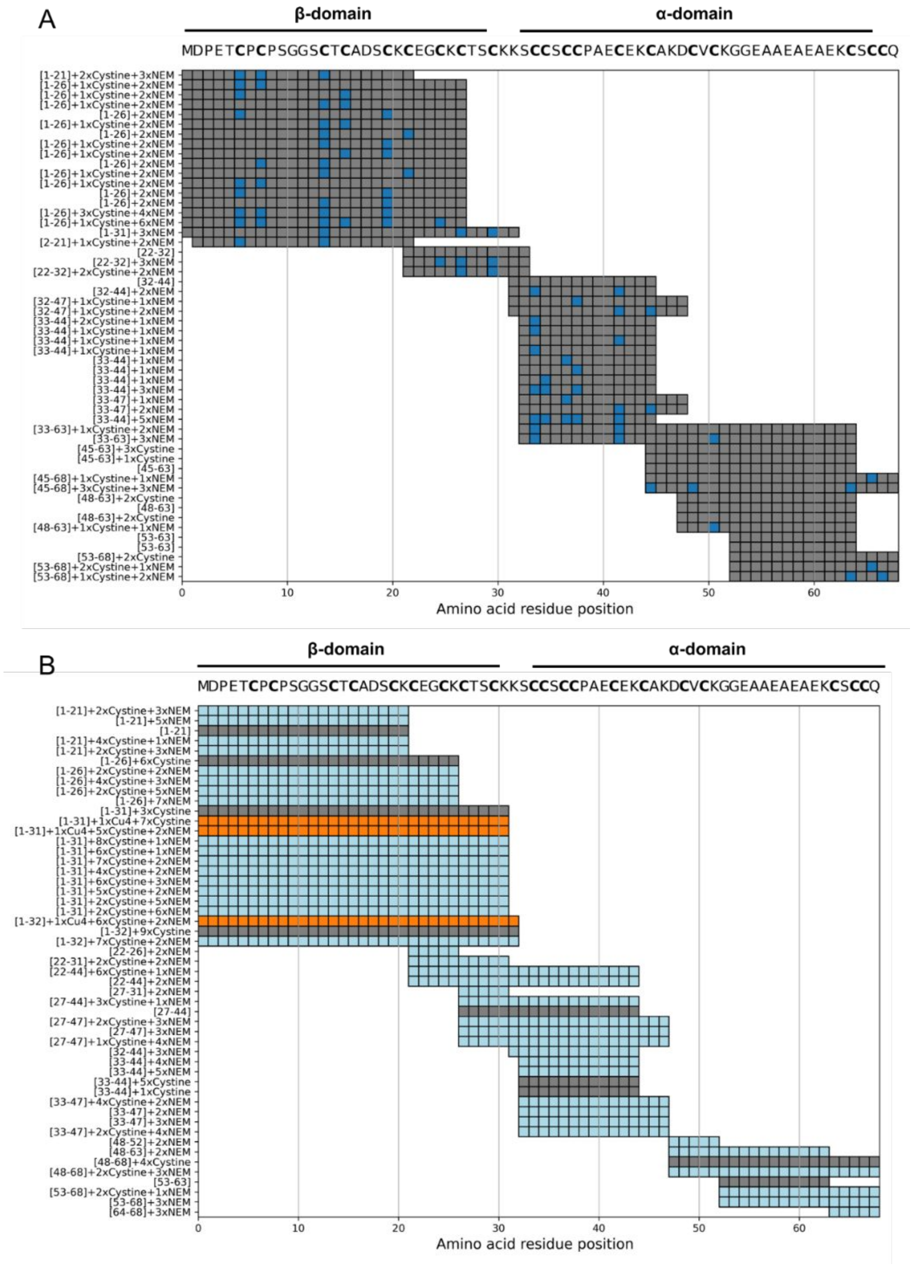

Figure S14. Bottom-up MS (LC-MS/MS) coverage maps (A) and peptide-mass fingerprint (B) for mapping Cys-NEM labeled residues in Cu(I)/Zn(I)-$MT3_{ox}$ complexes. The initial homogenous Zn_7MT3_{red} protein was incubated with 4 Cu(II) mol. Eq, and afterwards 25 mM NEM was added. The resulting heterogeneous labeled metal-protein complexes were digested into peptides and analyzed. In (A), the NEM-Cys labelled residues were coloured in blue. In (B), the peptides that modified by NEM were coloured by blue, while those that had Cu(I) bound were coloured in orange.

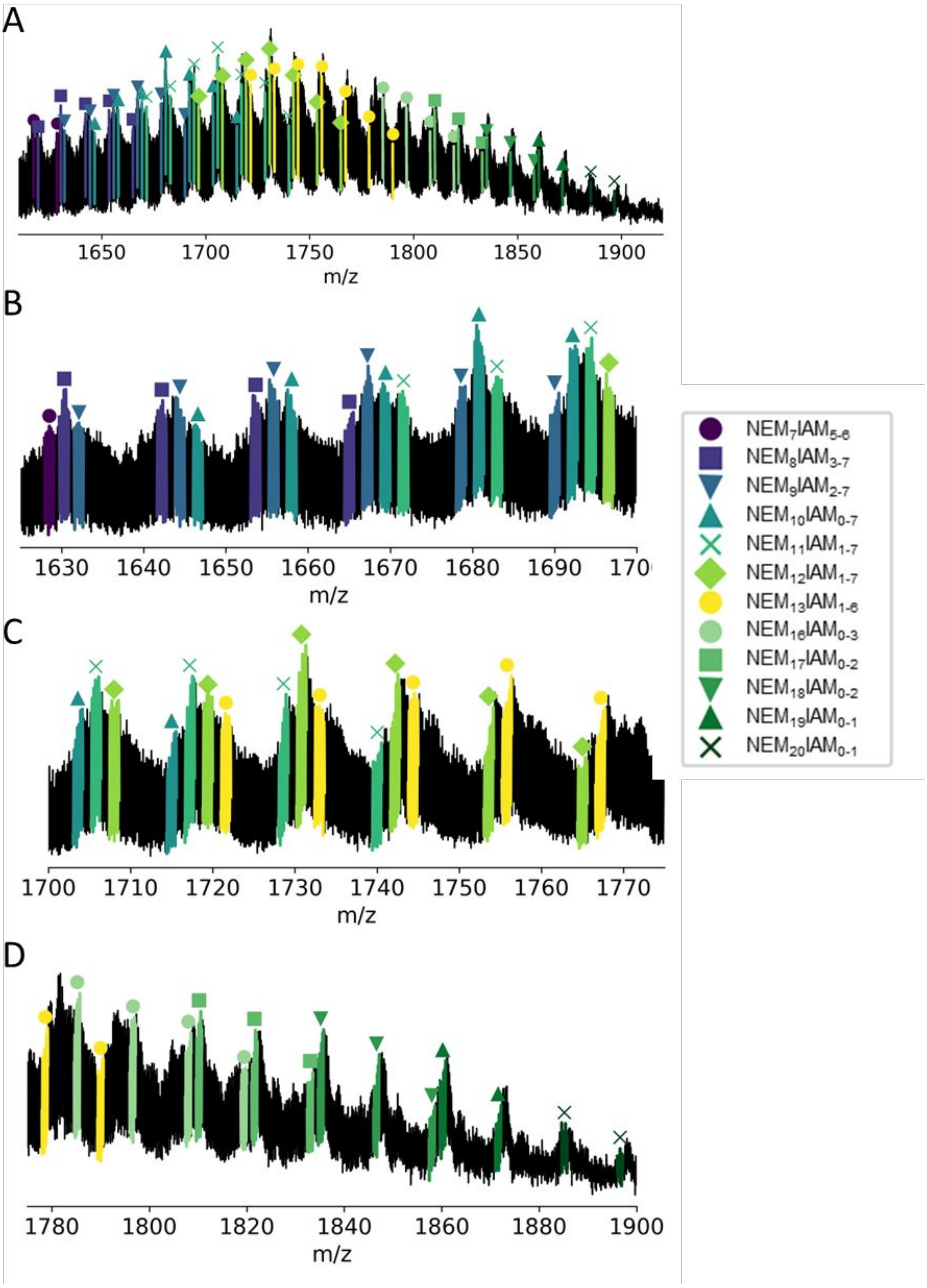

Figure S15. Native mass spectra of NEM- and IAM-Cys labeled residues of the Cu(I)/Zn(II)-MT3 complexes obtained after incubation of Zn_7MT3_{red} with 4 $CuCl_2$ mol Eq. The m/z region corresponds to 5+ ions (A), and specific *m/z* regions are zoomed in for clarity (B-D).

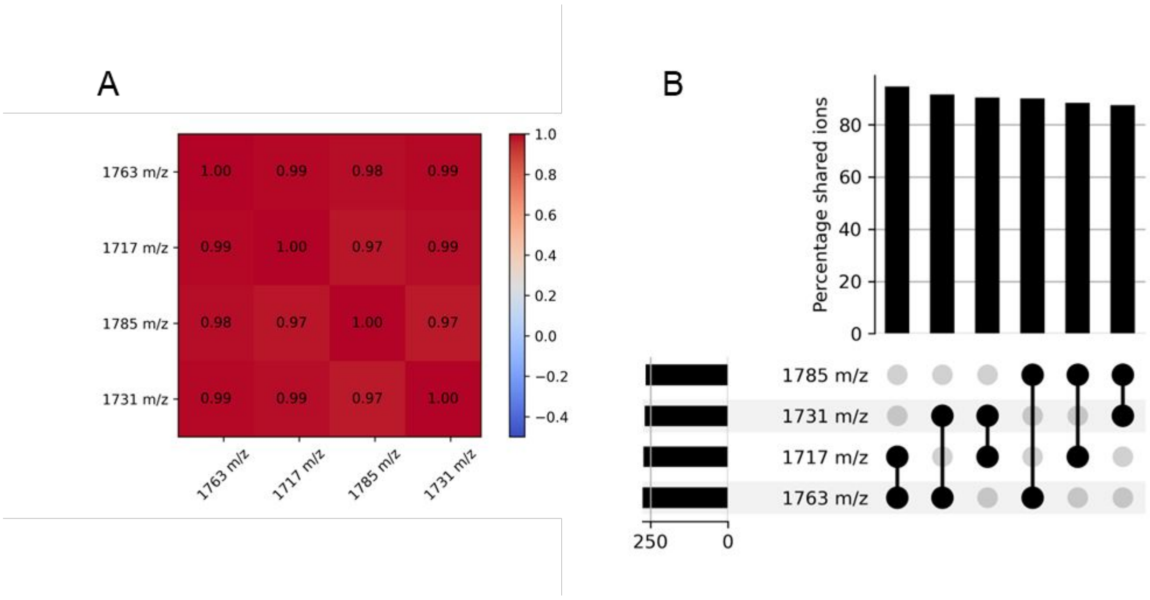

Figure S16. Analysis of the top-down CID MS for double IAM- and NEM-Cys labeled Cu(I)/Zn(II)-$MT3_{ox}$ complexes obtained after incubation of Zn_7MT3_{red} with 4 $CuCl_2$ mol Eq. Correlation matrix based on peak intensities of the fragmentation spectrum (A) and Upset plot analysis, which permitted us to visualize the percentage of shared ions in all fragmentation spectra (B). On the left, it is shown the set size that compose each fragmentation spectra (e.g. 250 detected peaks).

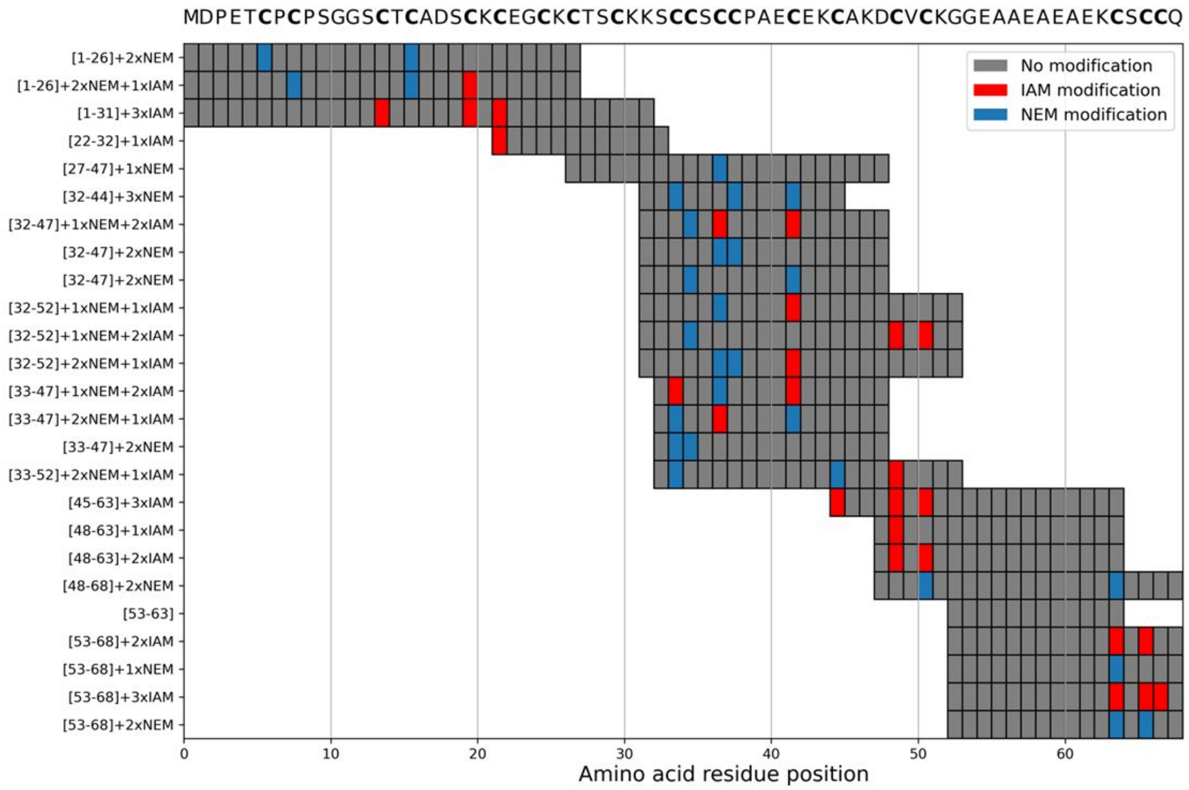

Figure S17. Bottom-up MS (LC-MS/MS) coverage maps for mapping Cys-NEM/IAM labeled residues in Cu(I)/Zn(I)-MT3 complexes. The initial homogenous Zn_7MT3_{red} protein was incubated with 4 Cu(II) mol Eq, and afterwards 25 mM NEM was added. The resulting mixture of species was then alkylated with 100 mM IAM, digested using trypsin, and analyze by LC-MS/MS.

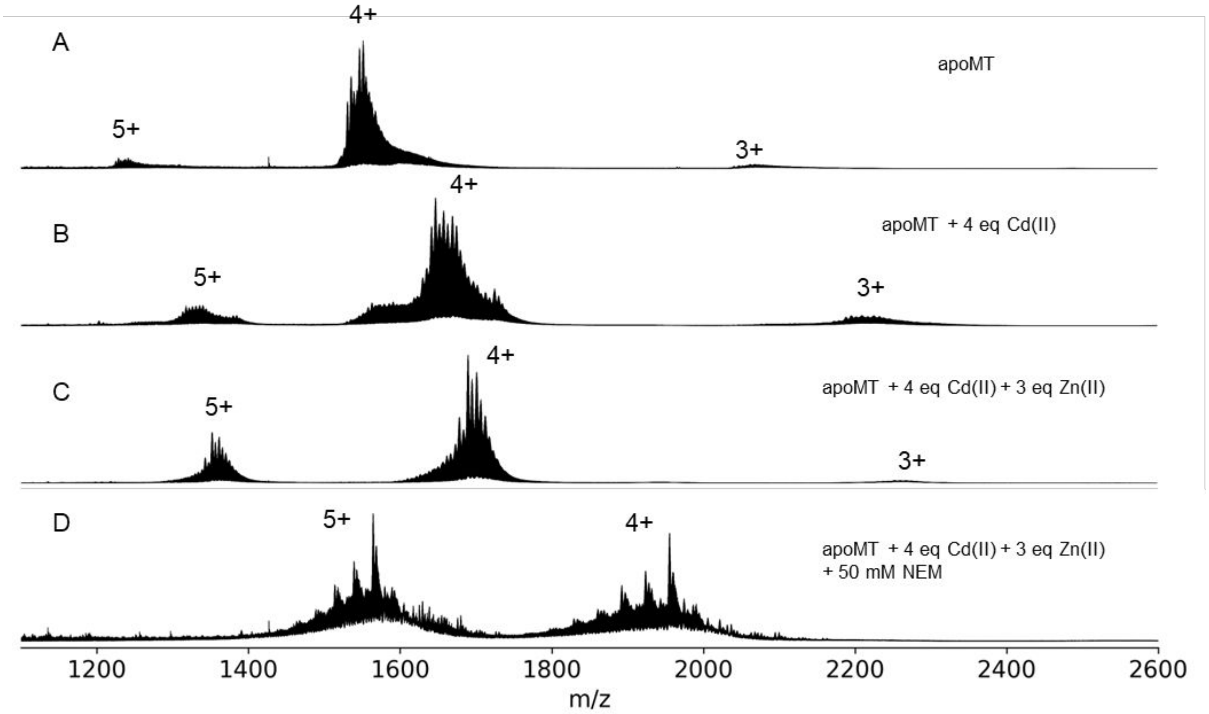

Figure S18. Native mass spectra of rabbit apo-metallothionein (A), after addition of 4 Cd(II) mol. Eq (B), 4 Cd(II) and 3 Zn(II) mol. Eq. (C) and, after incubation with 50 mM NEM (D).

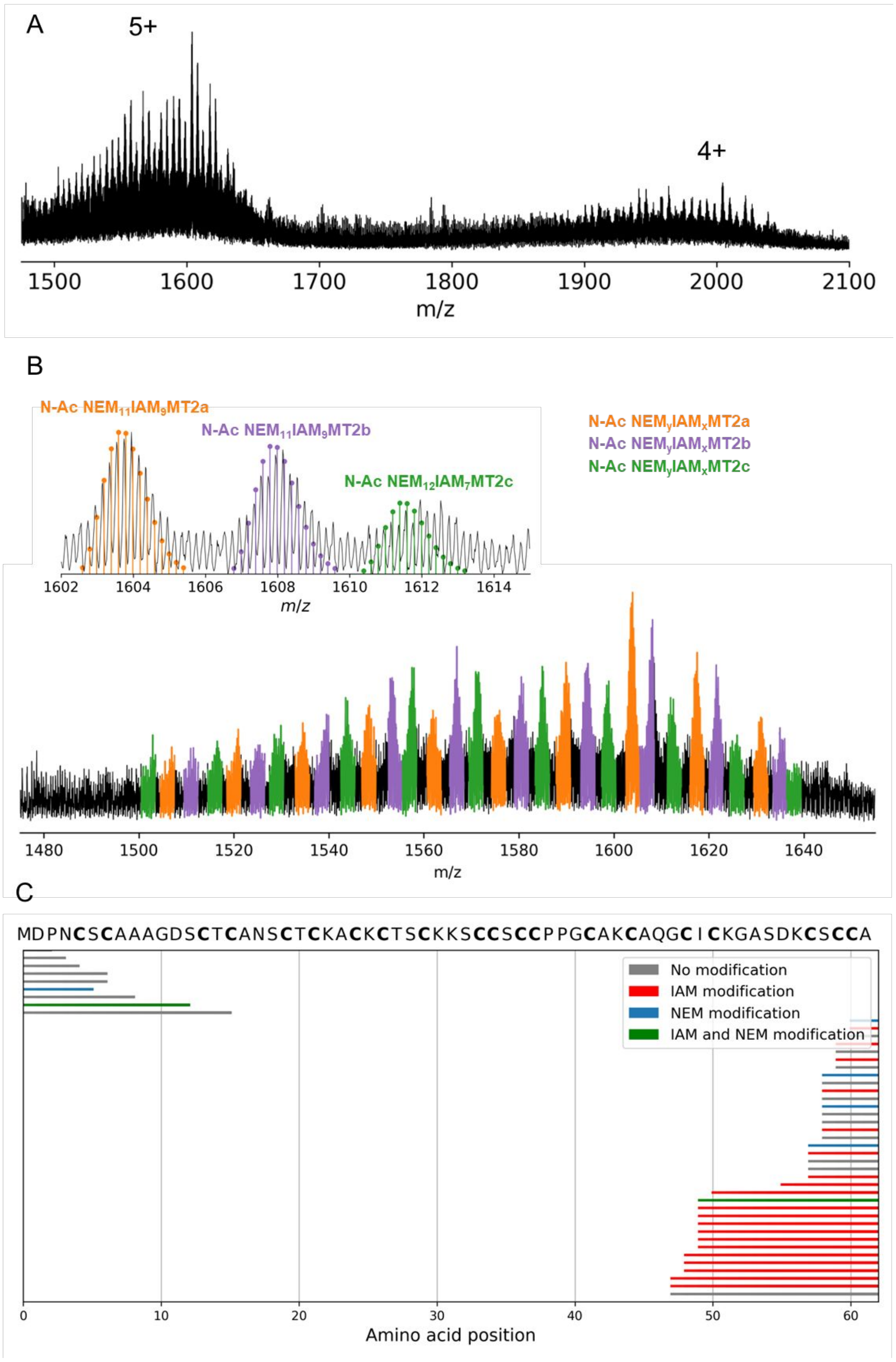

Figure S19. Native mass spectra of rabbit apo metallothionein after addition of 4 Cd(II) and 3 Zn(II) mol, and incubated with 50 mM NEM and 50 mM IAM (A). Zoom of the 5+ ions from (A) and isotopic fitting of some species. Top-down CID MS of quadrupole isolated $NEM_{11}IAM_9MT2a$ ions.

REFERENCES

1 Krężel, A.; Maret, W. The bioinorganic chemistry of mammalian metallothioneins. *Chem. Rev.* **2021**, *121*, 14594–14648.

2 Eyer, P.; Worek, F.; Kiderlen, D.; Sinko, G.; Stuglin, A.; Simeon-Rudolf, V.; Reiner, E. Molar absorption coefficients for the reduced Ellman reagent: reassessment. *Anal. Biochem.* **2003**, *312*, 224–227.

3 Peris-Díaz, M. D.; Guran, R.; Domene, C.; de los Rios, V.; Zitka, O.; Adam, V.; Krężel, A. An integrated mass spectrometry and molecular dynamics simulations approach reveals the spatial organization impact of metal-binding sites on the stability of metal-depleted metallothionein-2 species. *J. Am. Chem. Soc.* **2021**, *143*, 16486–16501.

4 Peris-Díaz, M. D.; Guran, R..; Zitka, O.; Adam, V.; Krężel, A. Metal- and affinity-specific dual labeling of cysteine-rich proteins for identification of metal-binding sites. *Anal. Chem.* **2020**, *92*, 12950–12958.

5 Gabelica, V.; DePauw, E. Internal energy and fragmentation of ions produced in electrospray sources. *Mass Spectrom. Rev.* **2005**, *25*, 566–587.

6 Virtanen, P. et al. SciPy 1.0: Fundamental algorithms for scientific computing in python. *Nat. Methods*, **2020**, *17*, 261–272.

7 Strohalm, M.; Kavan, D.; Novák, P.; Volný, M.; Havlíček, V. mMass 3: a cross-platform software environment for precise analysis of mass spectrometric data. *Anal. Chem.* **2010**, *82*, 4648–4651.
